# Assessment of Validity of Children's Movement Skill Quotient (CMSQ) Based on the Physical Education Classroom Environment

**DOI:** 10.1155/2020/8938763

**Published:** 2020-10-16

**Authors:** Jindong Chang, Yan Li, Hanbing Song, Liming Yong, Lin Luo, Zhanjia Zhang, Naiqing Song

**Affiliations:** ^1^School of Physical Education, Institute of Motor Quotient, Southwest University, Chongqing 400715, China; ^2^School of Mathematics and Statistics, Southwest University, Chongqing 400715, China; ^3^The Branch of the Collaborative Innovation Center of Assessment toward Basic Education Quality, Southwest University, Chongqing 400715, China; ^4^Key Laboratory of Physical Fitness Evaluation & Motor function Monitoring, General Administration of Sport of China, Southwest University, Chongqing 400715, China; ^5^School of Physical Education, Guizhou Normal University, Guiyang 550001, China; ^6^Department of Physical Education, Peking University, Beijing 100871, China

## Abstract

The development of movement skills in children is a critical element in promoting physical activity and other positive health trajectories over their lifetime. A reliable and valid assessment tool is essential for evaluating children's movement skills in daily physical education environments. The purpose of this study was to examine the validity of Children's Motor Skills Quotient (CMSQ) used in the classroom setting. Six raters conducted evaluation to participants, and a total of 734 children completed all the test items and were included in the study. Descriptive statistics and Rasch analysis were used in this study. The descriptive statistics were mainly used for calculating the mean, standard deviation, percentage, and internal consistency coefficient. Rasch analysis was used to verify the fitting statistics, project difficulty, and functional differences of the items of the CMSQ. The findings showed that the CMSQ met the assumption of the Rasch model, including the unidimensionality, local independence, person measure, and item difficulty hierarchy. The CMSQ also demonstrated adequate interrater reliability and internal consistency. The differential item functioning (DIF) demonstrated a few items showing different probabilities across sex and age. To maintain the item difficulty hierarchy of the CMSQ, no items were deleted. Overall, the CMSQ seems to have appropriate test items with an appropriate rating scale structure for measuring 6-9-year-old children's movement skills in the physical education classroom environment.

## 1. Introduction

Numerous studies demonstrate that sufficient participation in physical activity is beneficial for children's fitness and health [1–4]. Still, across the globe, more than 80% of children and adolescents fail to meet the physical activity recommendations of the World Health Organization (WHO) [5]. Some studies indicate that health-related physical fitness is positively correlated with the development of movement/motor skills in children [6–10]. Research also suggests that developing appropriate levels of motor skills in children may be a critical element in promoting their physical activity and other positive health trajectories over their lifetime [9, 11]. Recent literature emphasizes the importance of competence in movement skills concerning the positive developmental trajectories of children's health [11]. Today, the development of children's motor skills has become of paramount importance in promoting children's health and fitness [7, 9–11]. Evaluating the development of children's movement/motor skills is also a significant concern in the contemporary motor development field.

In the past decade, assessment has made tremendous achievements in the education system [12]. Large-scale international educational assessment projects such as the Program for International Student Assessment (PISA) and Trends in International Mathematics and Science Study (TIMMS) are widely used in the assessment of reading, mathematics, and science in children and adolescents [13–18]. As part of the education system, movement testing in physical education has also gained extensive attention [19], and various studies of movement testing in physical education have been conducted in recent years [20–26]. However, because of the inconsistent general academic terms and different research purposes, the concepts, operations, and measurements in the research of motor skills overlap and are confusing [9, 23, 27, 28]. The concepts of “motor skill,” “movement skill,” “motor skill competency,” and so on are used interchangeably to express the same meaning, but they form their own systems. Logan et al. reviewed the term “fundamental movement/motor skills” (FMS), and they found that fundamental movement skills (69%) are used more frequently than fundamental motor skills (31%) in the literature [25]. Considering that the term “movement” is more specific and widely used than “motor,” the following use of movement skill includes the meaning of motor skill and motor skill competency.

The primary purpose of the movement test in physical education is the identification of students with motor deficiencies or the description/monitoring of motor proficiency levels [29, 30]. The choice of an assessment tool and their reliability and validity is critical in assessing children's motor skills. Cools et al.'s study indicated that the most frequently used assessment tools for children aged 6–9 were the Movement Assessment Battery for Children (MABC) [31], the Test of Gross Motor Development (TGMD-2) [32], the Bruininks–Oseretsky Test of Motor Proficiency (BOTMP) [33], the Körperkoordinationstestfür Kinder (KTK) [34], and the Maastrichtse Motoriek Test (MMT) [35]. The frequency of use can be used as a reference indicator for the choice of children's movement skills assessment tools in the physical education classroom setting.

Satisfying reliability and validity are prerequisites for the selection of children's motor skills assessment tools. Scheuer et al.'s [26] literature review showed that the assessment tools of movement skills with the highest validity scores are the athletic skills track (AST) [36], the BOT-2, the MABC-2, the MAND-2 [37], and the TGMD-2. However, Scheuer et al. also found that the factorial validity of several test instruments has not been established and that more studies are needed to verify their psychometric quality for use in physical education testing in the future [26]. Cools et al. reviewed these different assessment tools and concluded that the concurrent validity of the majority of them was moderate [35], thus showing that they lacked feasibility in a physical education setting. Addressing the controversy over whether the assessments should be process- or product-oriented, Logan et al. supported using a combination of process and product assessment patterns, and choosing an assessment pattern based on the purpose of evaluation [25]. Ward et al. investigated the impact of assessor experience on assessment accuracy and stated that accurate criterion identifications are crucial in conducting motor skills assessment [24]. Therefore, reliability and validity should be satisfied not only for the assessment tool and the interraters but also for the assessment pattern and a physical education settings.

**Figure 1:**
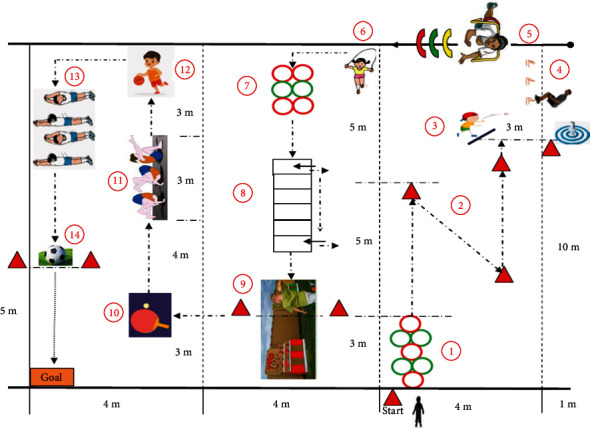
The flow figure of the CMSQ.

Despite some tools having acceptable reliability and validity and being suitable for the measurement and evaluation of movement skills, most of these tools are composed of several (e.g., the KTK with four items) to dozens (e.g., the MABC with 32 items) of independent movements with no sequential connection among them [31, 35]. Only a few movement skills assessment tools are composed of a continuous sequence of test combinations, such as the AST, the Canadian Agility and Movement Skill Assessment (CAMSA) [38], and the Children's Movement Skill Quotient (CMSQ) [39]. The AST consists of a series of fundamental motor tasks (*n* = 10) that include crawling, hopping, traveling, jumping, throwing and catching, kicking, front rolling, back rolling, walking backwards, climbing, and jumping [35]. It is a feasible screening tool in a physical education setting, as each child can complete each test in less than one minute. Moreover, sports equipment and materials for testing are readily available in every gym. The CAMSA was developed to assess the fundamental, combined, and complex movement skills of children to determine childhood physical literacy. It includes seven items and 14 scoring points, and each test should be completed within 30 seconds; otherwise, the score is recorded as 0 points [38]. The CAMSA test is suitable for children aged 8-12 years old. The CMSQ is first reported orally at the 66th Annual American College of Sports Medicine (ACSM, 2019) and is a sequential combination of movement skills, which includes 14 items (see Figure 1 and Table 1), each of which needs to be evaluated for processes and products, and the completion time is also recorded [39].

To perform tests, equipment and materials are required. The knowledge of the test protocols can be mastered by most teachers in the physical education curriculum, and the test cost should not be too high nor should the test time be too long. Cools et al.'s study showed that for the four typical instruments of independent movement (i.e., the M-ABC, the KTK, the TGMD-2, and the BOT-2), the test cost was in the range of €262–1352, and the assessment time varied from 20 to 60 minutes [35]. As such, considering the assessment time, cost, and level of the assessors, it was appropriate to develop the CMSQ as an assessment tool of the movement skills in the physical education classroom setting.

Product-based measures are the most common approach to assessing movement skills [40], and the Rasch model has been increasingly applied to determine the validity of motor test tools [41]. To the best of our knowledge, the CMSQ is the first children's movement skills assessment tool with a skill track in a physical education setting that measures process-based (i.e., skill level) and product-based (i.e., skill completion) scores, as well as completion time. The skill components, the completion time, and the weight of the skill score were determined by 19 international experts through the three-round experts Delphi's progress [39]. Although the CMSQ reported its face validity, the sample size (only 58 subjects) and statistical methods limited the validity of the CMSQ. The purpose of this study was to examine the validity of the CMSQ using Rasch analysis to evaluate the fit of the CMSQ items, to test for potential item bias, to check the dimensionality and rating scales, to determine the item difficulty, and to establish the interrater reliability.

## 2. Materials and Methods

### 2.1. Materials

The CMSQ is a part of the assessment of the Chinese Children Motor Quotient (CCMQ) developed by Chang et al. [39]. The CMSQ is a set of continuous, closed-loop, and combined motor skill test tools that were recommended and selected by 19 international experts. The CMSQ consists of 14 items in three types, including locomotor (e.g., running, hopping, and crawling), object control (e.g., catching, throwing, and kicking), and stability status (e.g., roll and balance) skills [39]. Its scoring system includes three sections: (1) process-oriented evaluation, (2) product-oriented evaluation, and (3) time evaluation. The present study explored the second part of the production-oriented evaluation, and Table 1 shows an explanation of the movement skills and rating standards. The product-oriented evaluation recorded a score of 0 points or 1 point. The children who participated in the test successfully were given 1 point for that skill; otherwise, the score was recorded as 0 points. The process-oriented evaluation record was 0, 1, and 2 points. The time was converted to 0-42 points in the corresponding order. The test flow is shown in Figure 1, and the details of the measurement process are introduced in Section 2.3.

### 2.2. Participants

This study was approved by the Ethics Committee of the Institute of Motor Quotient, Southwest University (IRB No: SWUIMQ20180312) and followed the guidelines of the International Declaration of Helsinki. All children were required to be healthy and free from congenital physical defects (e.g., physical disability). All participants with approval from their parents or guardians and who completed all the tests were included in this study. The participants included valid convenience samples of 734 children aged 6-9 (369 males and 365 females) from four cities (i.e., Beibei in Chongqing, Xuzhou in Jiangsu Province, Kunming in Yunnan Province, and Suining in Sichuan Province) in China. All participants were enrolled in public schools; one urban school and one rural school were selected from each city. The population of students in the candidate elementary schools was not less than 600. Participants were classified into four age groups: 6, 7, 8, and 9 years from grades 1 to 3. Twenty-five children were randomly selected from each age group to participate in the test. The demographic information of the participants is shown in Table 2.

### 2.3. Measures

#### 2.3.1. Rater Assessment

The tests in this project were carried out jointly by six raters. The raters were trained in the strict professional evaluation of the CMSQ for two weeks. In the process of training, we provided instructions and demonstrations of the CMSQ testing procedures and rating criteria, as well as specific skill analysis combined with videos, on-site presentations of action passwords and organizations, and practical operational guidance. Following the training, each rater was required to evaluate the CMSQ for twelve children (the number of evaluations during the training period was not included in the final validity analysis), including on-site evaluations for two children and video evaluations for 10 children. The consistency of the CMSQ scores was more than 0.8, which was considered acceptable. Finally, only the appraisers who passed the consistency check performed the tests.

#### 2.3.2. Measurement Procedure

The assessments were performed in a 15 × 15 meter square outdoor or indoor environment based on the specific site conditions of each school. In accordance with the CMSQ manual guidelines, the raters appraised the materials and tools in the CMSQ task for 6-9-year-old children. After setting up the test site, five nonselected children were invited to conduct a simulation test (only once). Each school was a test unit, and each test unit conducted a simulation test. The five invited kids were tested one by one before the formal test in each school. The primary purpose of these testing included three aspects: (1) to examine whether the site met the test standards, (2) to examine whether the cooperation between raters was consistent, and (3) to calibrate the consistency between raters. The participants were divided into groups of five children. Before the formal test, each group of children was given 10 minutes of video learning of the test content, and an evaluator was responsible for explaining the requirements of the CMSQ. After entering the test site, each child practiced once and then carried out the test. Each child was tested twice, taking the best score as the outcome.

In the trial, the six raters were divided into two groups. Rater A was responsible for organizing (i.e., video learning and management of the test site), rater B for guiding (i.e., issuing action passwords) and timing, and rater C for rating. Rater A needed to explain the test content to the children and to ensure they stayed quiet on the test site. Rater B was mainly responsible for guiding the children to conduct the action, issuing the action password, throwing and picking up the ball, and so on. This test did not check the children's memory. Therefore, rater B needed to issue a password directly, accurately, and in a timely manner in the test. Rater C was required to concentrate on each movement of the child and to judge it accurately according to the CMSQ standard guidelines. For each test group, the division of labor was rotated to ensure the accuracy of the raters' judgment. Each group of tests typically took about 45 minutes. Twenty groups were tested in each school, which took five days to complete. The test duration was six weeks, from May 6 to June 15, 2019. To ensure the relative consistency of the test results, we considered weather factors to determine the test arrangements. All tests were conducted in good weather conditions, with indoor or outdoor temperatures between 20 and 30°C.

### 2.4. Statistical Analysis

Descriptive statistics were performed for participants' characteristics, such as gender (*N*, %), age (mean ± standard deviation (SD)), height (mean ± SD), weight (mean ± SD), and sample sources (*N*, %). The intraclass correlation coefficient (ICC) was used to examine the interrater reliability, and values greater than 0.80 were considered satisfactory [42]. The internal consistency was analyzed using Cronbach's alpha, and the values of more than 0.70 were acceptable [43]. SPSS19.0 software (SPSS Inc., Chicago, USA) was used to perform the above statistical analysis.

Rasch analysis was performed to verify the internal validity and the level of difficulty of the items in the CMSQ using the Winsteps software, version 4.5.4 (Oregon, USA). The unidimensionality was examined using principal component analysis (PCA) of the Rasch residuals. Once the product-based measures of the CMSQ showed a unidimensionality measurement structure, an item-level analysis was used to perform the Rasch analysis, including rating scale analysis, item fit statistics, person response validity, item difficulty, and differential item functioning (DIF) [44].

#### 2.4.1. Unidimensionality

The core assumption of the Rasch model is to verify the single dominant measurement constructs in test items. A PCA of the Rasch residuals was used to examine the unidimensional constructs of the product-based measures of the CMSQ items [45, 46]. The unidimensionality assumption for the PCA of the Rasch residuals was satisfied by the following criteria: (1) the variance was explained for more than at least 40% by the measurement dimension [47, 48], (2) the variance explained by the first construct was less than 10%, and (3) the eigenvalue of the first construct was less than 2.0 [49].

#### 2.4.2. Rating Scale Analysis

The rating scale model (RSM) was used to measure the rating scale structure. The RSM was selected to calculate the parameters based on the two response options of the product-based measures of the CMSQ [50]. The following criteria verified the fit of the rating scale to the Rasch model. First, at least 10 observations were made in each response category. Subsequently, the outfit mean squares (MnSq) for rating scale categories were less than 2.0, and monotonically advanced average measures could be observed in the rating scale categories [51]. If most of these criteria were not met, the rating categories were reorganized (i.e., some categories were collapsed) [52].

#### 2.4.3. Item Fit Statistics

Fit statistics were performed to monitor the compatibility of the raw item data with the expectations of the Rasch model. Two types of fit statistics showed that the infit statistic (weighted information) was the most sensitive to ratings on the item difficulty close to the children's ability, whereas the outfit statistic (unweighted information) was more affected by the scores of the off-target items. The infit and outfit MnSq and standardized *z* values (Zstd) were used to test the fit of the Rasch model [53]. The fit criteria of MnSq values were more than 0.7 and less than 1.3, and the ZSTD values were within −2.0 and 2.0 [54].

#### 2.4.4. Item Difficulty

In the motor test, the item difficulty refers to the rate at which children demonstrated motor skills correctly: the higher the skill difficulty, the lower the correct execution rate, demonstrating the test's suitability. In the Rasch model, item difficulties and personal ability were located on the same linear continuum (logits), and an item–person map presented the matching between test items and person measures. In the process of analysis, it was examined whether the order of item difficulty levels estimated by the Rasch model was consistent with the logical progression from the simplest to the most difficult [55]. The ceiling/floor effects and possible gaps (i.e., a certain ability level was low or no items existed) were examined, as well as whether the ceiling and floor impact criteria exceeded 5% of the sample in the maximum and minimum person measures [56].

#### 2.4.5. Differential Item Functioning

DIF was used to estimate the Rasch-based item difficulty parameters [54, 57]. The Rasch–Welch *t*-test was used to verify the magnitude of DIF [58]. On the map, the individuals were identified with DIF by gender (male vs. female) and age type (6 years old vs. 7 years old, 7 years old vs. 8 years old, and 8 years old vs. 9 years old) [59]. The effect size of DIF was calculated using the following criteria: (1) a moderate-to-large DIF (more than 0.64 logits, indicating a difference in the comparison groups) and (2) a slight-to-moderate DIF (more than 0.43). An alpha value of 0.05 with a two-sided Rasch–Welch *t*-test was used to determine the significance of DIF [58].

## 3. Results

### 3.1. Interrater Reliability and Internal Consistency

The ICC of the product-based measures of the CMSQ was 0.96 (95% confidence interval (CI) = 0.94-0.98), indicating that the interrater reliability was high. Meanwhile, the internal consistency (Cronbach's alpha = 0.80) of the14 items of the CMSQ was acceptable.

### 3.2. Unidimensionality

The PCA of the Rasch residuals displayed that 42.7% of the variance was explained by the Rasch model, and the eigenvalue of the first construct was 1.5 (6.2%), while that of the second was 1.3 (5.4%). Next, Rasch analysis of the CMSQ was conducted based on the results of the factor analysis. The residual correlation matrix of a single dominant factor showed that there was no violation of the local independence assumption in the test items (*r* = 0.021 − 0.372).

### 3.3. Rating Scale Analysis

The rating scale analysis results showed that the 14 items of the CMSQ had good fit. All items' MnSq values (outfit MnSq = 0.74-1.15) were between the standard values in the range of 0.7-1.3. Thus, no rating scale reorganization was made.

### 3.4. Item Fit

Table 3 shows the item fit results of the Rasch model. The items with infit MnSq values ranged from 0.90 to 1.19, while the outfit MnSq values were from 0.74 to 1.15. In the MnSq values, there were no overfitted or misfitted items, while the infit Zstd values of Item CS05 (3.53) and Item CS14 (2.94) were greater than 2.0. The outfit Zstd value of Item CS08 was less than -2. In terms of Zstd values, Items CS05, CS08, and CS14 did not meet the requirements of the criteria set. However, the Winsteps guidelines state that the infit and outfit values must be in the range of 0.5-1.5; the values of Zstd are for reference only [58]. Therefore, the overall fit statistics of the product-based measures of the CMSQ illustrated a good fit.

### 3.5. Item Difficulty

**Figure 2:**
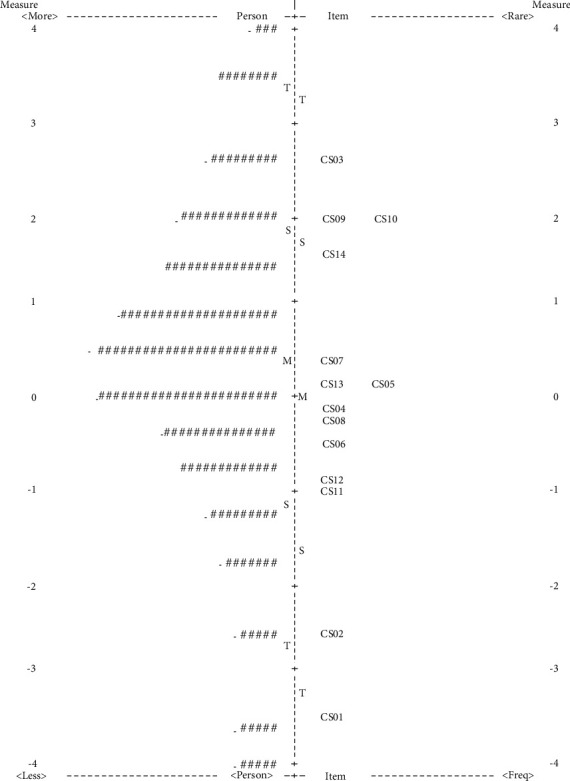
Person–item map for the CMSQ. Each (#) in the person column represents four people, while each (.) represents 1-3 people. More: high personal ability; less: low personal ability; rare: high item difficulty; less: low item difficulty; +M: item mean; M: person mean; S: 1SD from the mean; T: 2SD from the mean.

Figure 2 displays a person–item map of the personal abilities (logits measures) and item difficulties (distributions) for the 14 items of the CMSQ. On the map, more represents high personal ability, and less represents low personal ability. Rare represents high item difficulty and freq (frequent) represents low item difficulty. The most difficult items were Item CS03 (catching and throwing; 2.58 logits), Item CS09 (overhand throwing; 1.97 logits), and Item CS10 (bouncing; 1.95 logits), and the easiest items were Item CS01 (–3.47 logits) and Item CS02 (–2.59 logits). The mean of the personal abilities and item difficulties was located within 0.5 logits (person = 0.25 logits; item = 0 logits). The means logit measures of the participants in the CMSQ were found to be without significant deviation (item = 0.11 logits). Furthermore, no floor (2.9%) or ceiling (1.8%) effects were found in the person measure of the CMSQ.

In addition, the Rasch analysis revealed that the person reliability coefficient was 0.80 and the separation index was 1.97, while the item reliability coefficient was 1.00 and the separation index was 15.02. The study results indicate that the person and item measures can be reliably estimated at levels higher than 0.80 and 0.9, respectively [48].

### 3.6. Differential Item Functioning

DIF analysis found that four items fell outside of the criteria across sex and age (see Table 4). Item CS02 had a moderate DIF across sex (DIF contrast = −0.62, *p* = 0.04); Item CS01 (DIF contrast = −1.12, *p* = 0.01) and Item CS10 (DIF contrast = −0.83, *p* = 0.01) had a significant DIF by age (6 years old vs. 7 years old); and Item CS01 (DIF contrast = 1.15, *p* = 0.03) and Item CS12 (DIF contrast = −0.57, *p* = 0.04) also had a significant DIF by age (7 years old vs. 8 years old) (see Table 5). The DIF results indicate that children carrying out the movements associated with these items experienced different levels of difficulty depending on their sex and age.

## 4. Discussion

The primary purpose of this study was to examine the validity of the CMSQ using Rasch analysis. The study findings indicated that the product-based measures of the CMSQ seemed to indicate appropriate test items with an appropriate rating scale structure for measuring 6-9-year-old children.

As we expected, the CMSQ demonstrated adequate interrater reliability and internal consistency. The reliabilities between the raters obtained high values. However, the reliability analysis between the raters included only 12 subjects, which is much less than the minimum requirement of 50 [60]. The product-based measures of the CMSQ met the assumption of the Rasch model, including unidimensionality and local independence. The rating scale of the CMSQ displayed a good fit. The MnSq value of the infit and outfit was within a standard range, and there were no overfitted or misfitted items. However, the three items in the infit and outfit Zstd values exceeded standard values. An explanation given in the Winsteps manual is that if the MnSq value is in the standard range of 0.5-1.5, a Zstd value exceeding the limit can be ignored [40]. In this study, the MnSq values were between 0.74 and 1.19, and the overall item fit statistics were satisfactory.

The items of the CMSQ demonstrated a logical item difficulty hierarchy. In the person–item map, Item CS03 (catching and throwing; 2.58 logits), Item CS09 (overhand throwing; 1.97 logits), and Item CS14 (kicking; 1.95 logits) were the most difficult items, whereas Item CS01 (hoop jumping; –3.47 logits) and Item CS02 (sliding; –2.59 logits) were the easiest items. These results indicate that complex hand and foot movement skills were the most difficult tasks for 6-9-year-old children. Furthermore, we found that there were two groups of items with a similar difficulty level, namely, Item CS09 (overhand throwing; 1.97 logits) and Item CS14 (kicking; 1.95 logits), as well as Item CS13 (longitudinal roll; 0.18 logits) and Item CS05 (crawling; 0.15 logits). Other research proposes that similar difficulty levels of test items may not provide more calibration information for evaluating movement skills [44]. The best solution was to delete some of the items of the same difficulty levels. Determining whether to delete items has often been used as the internal consistency coefficient: If the internal consistency coefficient is too high (i.e., Cronbach's alpha was greater than 0.98), item redundancies are suggested [44] and it is considered necessary to delete some items. In this study, the internal consistency coefficient of the CMSQ was only 0.80; thus, we did not delete any items.

The items of the CMSQ demonstrated a good match with the person measure. The person reliability and person separation index values for the CMSQ were acceptable. No floor or ceiling effects were found in the person–item map, indicating that the items of the product-based measures of the CMSQ were able to distinguish children with high and low movement skills. The research by Wand et al. showed that the tools of the performance-based (process) assessment had a high level of prediction compared to self-reported measures [61]. In contrast with the process-based measures, during which children's performance outcomes were assessed (i.e., distance, speed, and accuracy) [40], the test results were normative data based on standards (i.e., success = 1 and failure = 0). In other words, in the product-based, process-based, and self-reported measures, the product-based measures had the highest precision, while the self-reported measures had the lowest. Furthermore, a good match between item difficulty and personal ability might reduce measurement errors in the process of person measures [44, 54]. When the distance between the mean of person measures and the mean of item difficulty is less than 0.5 logits, it is considered a good measurement. Indeed, the distance between the mean of person measures and item difficulties was 0.25 logits in this study.

The DIF results demonstrated only one item (i.e., CS02) showing a moderate DIF across sex, while three items displayed significant DIF across age (6 years old vs. 7 years old and 7 years old vs. 8 years old). A significant DIF was not found across age for 8 years old vs. 9 years old. Considering that there were sex differences in the development of children's motor skills, the DIF item across sex was not eliminated from the CMSQ in this study. Comparing the DIF items across ages, we found that Item CS01 had the lowest item difficulty level, and that Item CS10 and Item CS12 had the highest difficulty level. To maintain the difficulty level of the CMSQ test, these three items were not deleted.

The current study had three limitations. First, interrater reliability needed to be further explored, with at least 50 subjects being evaluated by each rater. Second, it was not sufficient that the validity of the CMSQ was assessed only by product-based measures. A comparative follow-up study of process-based measures should be conducted to determine whether the DIF items should be retained or deleted. Third, this study lacked a standard validity test for calibration. The CAMSA or AST should be used as a calibration tool in further studies to further verify the validity of the CMSQ.

## 5. Conclusions

The CMSQ demonstrated good validity indicators, including dimensionality, fit statistics, DIF, and item difficulty hierarchy. Although a few items did not fully satisfy the fitting, most of the indicators met the Rasch model verification standards. Thus, the product-based measures of the CMSQ were preliminarily proven to be valid. The CMSQ might be a feasible alternative motor test to assess the movement skills of children aged 6–9. In the future, the outcomes of process-based measures should be included to verify the reliability and validity of the CMSQ.

## Figures and Tables

**Table 1:** The skill description and rating standard of Children's Movement Skill Quotient (CMSQ).

Item	Name	Skill description	Rating standard (product)
CS01	Hoop jumping	Two-footed jump into a single hoop, the alternate jumps into a circle with each foot	No stepping on or missing circles
CS02	Sliding	Hands and feet in the same direction, sliding sideways, with hands touching the cone	The hand should not miss and the foot should not touch the cone
CS03	Catching and throwing	The tester throws the ball to the subject, the subject then catches the ball with both hands and underhand throws it to the target	Throw the ball to the target area
CS04	Hurdle jumping	Jump over three hurdles (height = 30 cm) without alternating feet	No stepping on or touching the bar and no changing feet
CS05	Crawling	Crawl forward, alternately with arms and legs, close to the ground through three 60-cm-high hurdles	Crawl on hands and feet and no touching the fence
CS06	Rope jumping	With feet together, swing the rope into place and jump 10–15 times	Continuous rope skipping without interruption
CS07	One-foot jumping	Jump with one foot over all of the hula hoops in turn	No stepping on or missing circles
CS08	Agility running	Move sideways to the left, placing feet alternately in and out of the agility ladder grid while moving forward	No stepping on the line and no missing the grid
CS09	Overhand throwing	After taking a ball, turn the lead arm and throw the softball at the target from above the shoulder	Hit the target area
CS10	Bouncing	Use a racket to bounce a table tennis 5–10 times	The ball should be bounced uninterrupted
CS11	Front rolling	Roll forward by crouching, bowing head, narrowing chest, tucking in stomach, and rolling over	Roll straightforward
CS12	Dribbling	Dribble a ball in place 10 times with one hand	This should be uninterrupted
CS13	Longitudinal rolling	With hands above the head, bringing the hands, feet, and torso, and then the hands, feet, and body inline, carry out a controlled body rollover 1.5 m with no change in posture	Straight-line rolling, and the slope should not exceed 15 degrees
CS14	Kicking	Running, supporting, swinging, kicking	Score a goal

Note: for each item, all the actions that met the rating standard were scored as 1, while any that did not were scored as 0.

**Table 2:** Demographic characteristics (*N* = 734).

Characteristics	Mean (SD)	*N* (%)
Age		
6 years old		196 (26.7)
7 years old		189 (25.7)
8 years old		174 (23.7)
9 years old		175 (23.8)
Gender		
Male		369 (50.3)
Female		365 (49.7)
Height		
6 years old	119.1 (4.2)	
7 years old	126.1 (3.6)	
8 years old	131.2 (4.5)	
9 years old	137.1 (5.6)	
Weight		
6 years old	22.2 (3.6)	
7 years old	25.9 (3.9)	
8 years old	28.6 (4.8)	
9 years old	32.7 (5.6)	
Sources		
Beibei, Chongqing		192 (26.2)
Xuzhou, Jiangsu		187 (25.5)
Kunming, Yunnan		171 (23.3)
Suining, Sichuan		184 (25.1)

Note: SD: standard deviation.

**Table 3:** Item fit statistics and item difficulty results.

Items	Measures (logits)	Model	Infit		Outfit	
		SE	MnSq	Zstd	MnSq	Zstd
CS03	2.58	0.12	0.92	–1.17	0.78	–1.22
CS09	1.97	0.10	0.97	–0.61	0.96	–0.20
CS10	1.95	0.10	0.90	–1.83	0.74	–1.72
CS14	1.55	0.10	1.17	3.53	1.13	1.02
CS07	0.36	0.09	0.94	–1.57	0.93	–0.89
CS13	0.18	0.09	0.94	–1.55	1.00	–0.01
CS05	0.15	0.09	1.11	2.94	1.15	1.79
CS04	–0.07	0.09	1.06	1.56	1.11	1.29
CS08	–0.21	0.09	0.92	–1.95	0.83	–2.14
CS06	–0.52	0.09	1.07	1.63	0.96	–0.36
CS12	–0.88	0.10	0.93	–1.36	0.88	–1.14
CS11	–1.00	0.10	0.97	–0.55	0.91	–0.73
CS02	–2.59	0.14	1.05	0.53	0.80	–0.83
CS01	–3.47	0.18	1.19	1.41	1.12	0.46

Note: MnSq: outfit mean squares; Zstd: standardized *z* values; SE: standard errors of the measures.

**Table 4:** Differential item functioning across sex and age.

Item	Sex (male= Ref. vs. female)					Age (6 years old = Ref. vs. 7 years old)				
	DIF	Joint	Rasch–Welch			DIF	Joint	Rasch–Welch		
	Contrast	SE	t	df	Prob.	Contrast	SE	t	df	Prob.
CS01	–0.13	0.37	–0.36	692	0.72	–1.12	0.45	–2.50	345	0.01^b^
CS02	–0.62	0.29	–2.11	672	0.04^b^	–0.13	0.38	–0.34	359	0.73
CS03	0.00	0.23	0.00	697	1.00	0.31	0.36	0.86	354	0.39
CS04	0.00	0.18	0.00	697	1.00	–0.28	0.25	–1.12	359	0.26
CS05	–0.28	0.18	–1.55	697	0.12	0.18	0.25	0.72	359	0.47
CS06	0.13	0.19	0.70	697	0.48	0.03	0.26	0.11	359	0.91
CS07	0.18	0.18	1.01	697	0.31	0.21	0.25	0.87	359	0.39
CS08	0.12	0.18	0.64	697	0.52	–0.05	0.25	–0.20	359	0.84
CS09	0.21	0.21	1.02	695	0.31	0.02	0.30	0.08	359	0.94
CS10	–0.25	0.20	–1.23	697	0.22	–0.83	0.30	–2.81	354	0.01^b^
CS11	–0.34	0.20	–1.71	695	0.09	–0.10	0.27	–0.38	359	0.70
CS12	0.00	0.20	0.00	697	1.00	0.33	0.27	1.23	359	0.22
CS13	0.17	0.18	0.94	697	0.35	0.22	0.25	0.90	359	0.37
CS14	0.25	0.19	1.29	696	0.20	0.38	0.27	1.41	357	0.16

Note: DIF: differential item functioning; t: t-statistic; df: degrees of freedom; ^b^*p* < 0.05; SE: standard errors of the measures.

**Table 5:** Differential item functioning across age groups (7 years vs. 8 years; 8 years vs. 9 years).

Item	Age (7 years old= Ref. vs. 8 years old)					Age (8 years old= Ref. vs. 9 years old)				
	DIF	Joint	Rasch-Welch			DIF	Joint	Rasch-Welch		
	Contrast	SE	t	df	Prob.	Contrast	SE	t	df	Prob.
CS01	1.15	0.51	2.24	295	0.03^b^	0.13	0.69	0.19	317	0.85
CS02	–0.25	0.39	–0.64	348	0.52	0.22	0.44	0.50	322	0.62
CS03	–0.04	0.34	–0.13	348	0.90	0.41	0.32	1.31	325	0.19
CS04	–0.05	0.25	–0.20	347	0.84	–0.09	0.26	–0.36	335	0.72
CS05	0.38	0.25	1.52	346	0.13	–0.26	0.26	–0.98	335	0.33
CS06	0.12	0.26	0.46	345	0.64	–0.21	0.28	–0.76	334	0.45
CS07	0.08	0.25	0.34	347	0.74	–0.13	0.26	–0.51	335	0.61
CS08	–0.02	0.25	–0.08	347	0.94	0.02	0.27	0.08	334	0.94
CS09	0.06	0.29	0.22	348	0.83	–0.18	0.28	–0.62	335	0.53
CS10	0.38	0.30	1.26	348	0.21	–0.17	0.29	–0.61	335	0.54
CS11	–0.05	0.27	–0.16	346	0.87	0.21	0.30	0.68	329	0.50
CS12	–0.57	0.27	–2.08	348	0.04^b^	0.49	0.29	1.67	325	0.10
CS13	–0.11	0.25	–0.46	347	0.65	0.49	0.26	1.85	333	0.07
CS14	–0.41	0.27	–1.56	345	0.12	–0.47	0.28	–1.69	335	0.09

Note: DIF: differential item functioning; t: t-statistic; df: degrees of freedom; ^b^*p* < 0.05; SE: standard errors of the measures.

## Data Availability

The datasets analyzed during the current study are available from the corresponding author on reasonable request.

## Supplementary Materials

Supplementary Materials Table S1: the data of CMSQ (product). Video S1: the test video of the CMSQ. Video S2: rater assessment video of the CMSQ.
