# Supplementary material for: Assessment of Validity of Children's Movement Skill Quotient (CMSQ) Based on the Physical Education Classroom Environment
Source: Biomed Res Int. 2020 Oct 16;2020:8938763. doi: 10.1155/2020/8938763 (PMC7586154; doi:10.1155/2020/8938763)
Supplement: Supplementary Materials — Table S1: the data of CMSQ (product). Video S1: the test video of the CMSQ. Video S2: rater assessment video of the CMSQ. [file 8938763.f1.zip › Table S1 Data of CMSQ(Product).pdf]

id	sex	age	source	height	weight	CS01	CS02	CS03	CS04	CS05	CS06	CS07	CS08	CS09	CS10	CS11	CS12	CS13	CS14
CY001	1	8	1	134.5	26	1	1	0	0	1	1	1	1	0	0	1	1	1	0
CY002	1	9	1	141	35	1	1	0	0	1	1	0	0	0	1	1	1	1	0
CY003	2	9	1	141.5	27.5	1	1	1	0	1	1	1	1	1	0	0	1	1	0
CY004	2	9	1	135	30	1	1	0	0	0	0	0	1	0	0	1	1	1	0
CY005	2	9	1	130	32.5	1	1	0	0	1	1	1	0	0	1	1	1	1	1
CY006	1	7	1	126	27	1	1	0	0	1	1	0	0	1	0	1	1	1	0
CY007	2	9	1	135.5	30	1	1	0	1	1	1	1	0	1	1	1	1	1	0
CY008	1	9	1	138	31	1	1	1	1	1	1	1	1	1	0	1	0	1	0
CY009	2	9	1	136.5	33.5	1	1	0	0	0	1	0	0	0	0	1	1	1	0
CY010	2	8	1	133.5	26	1	1	0	1	0	0	1	1	0	1	1	1	1	1
CY011	2	9	1	134.5	33	1	1	0	0	1	1	1	1	0	0	1	1	1	1
CY012	2	9	1	131.5	30.5	1	1	0	0	1	1	1	1	1	0	1	1	1	0
CY013	1	8	1	130.5	26	1	1	0	0	1	0	1	1	1	0	0	1	1	0
CY014	2	7	1	129	30.5	1	1	0	1	0	1	0	1	0	0	1	1	1	0
CY015	2	8	1	128.5	25.5	1	1	0	0	1	1	0	0	1	0	0	0	1	1
CY016	2	9	1	131	27	1	1	0	1	1	1	1	1	1	0	1	1	1	0
CY017	1	9	1	129	24	1	1	0	0	1	1	1	1	0	0	0	1	1	0
CY018	2	9	1	130	23.5	1	1	1	1	1	1	1	1	1	1	1	1	1	0
CY019	1	7	1	132.5	28	1	1	0	1	1	0	1	1	1	1	1	1	1	0
CY020	1	9	1	136	35	1	1	0	1	1	0	1	0	1	1	1	0	1	0
CY021	2	9	1	133.5	30.5	1	1	1	1	0	0	1	1	1	0	1	1	1	0
CY022	2	8	1	129	23	1	1	1	0	1	1	0	0	0	0	1	1	1	0
CY023	1	8	1	134.5	37.5	1	1	0	0	0	1	1	1	0	0	1	0	1	0
CY024	1	8	1	126	25	1	1	0	0	1	0	0	1	1	0	1	0	1	1
CY025	2	8	1	126	23.5	1	1	0	0	1	1	0	0	0	0	0	1	0	0
CY026	1	7	1	125.5	32	1	1	1	1	0	1	1	1	0	1	1	1	1	0
CY027	1	9	1	133.5	29	1	1	1	0	1	0	0	0	0	0	1	1	0	0
CY028	1	7	1	123	28	1	1	1	0	1	0	1	0	0	0	1	1	1	0
CY029	1	6	1	122.5	23	1	1	0	0	0	0	1	1	1	0	1	1	1	1
CY030	1	7	1	128.5	22	1	1	1	1	0	0	0	1	0	0	1	1	1	0
CY031	2	7	1	126	22	0	1	0	1	0	0	1	0	0	0	0	1	1	1
CY032	1	8	1	129.5	28	1	1	0	0	1	0	1	1	1	1	1	1	1	0
CY033	2	8	1	132.5	29.5	1	1	0	1	0	1	1	1	0	1	1	1	0	1
CY034	2	9	1	132.5	24	1	1	0	1	1	1	1	1	1	1	1	1	1	0
CY035	2	7	1	129.5	31	1	1	0	1	1	0	1	1	1	0	0	1	1	0
CY036	2	9	1	131.5	27.5	1	1	0	1	1	1	1	1	0	0	1	1	1	0
CY037	2	6	1	126	28	1	1	1	1	0	0	1	1	0	0	0	1	1	0
CY038	2	9	1	133.5	34	1	1	0	1	1	1	1	0	0	0	1	1	1	0
CY039	1	6	1	119.5	21.5	1	1	0	1	0	1	1	1	0	0	0	1	1	0
CY040	2	8	1	125	26.5	1	1	0	1	1	1	1	1	0	0	1	1	1	0
CY041	1	9	1	143	30	1	1	0	1	1	1	1	1	0	0	1	1	1	0
CY042	2	9	1	125.5	23.5	1	1	0	0	0	1	0	1	0	0	1	1	1	0
CY043	2	6	1	120	22.5	1	1	0	1	1	0	0	1	1	1	0	1	1	0
CY044	2	9	1	141.5	32	1	1	1	0	0	0	1	0	1	1	1	1	1	0
CY045	1	8	1	138	35	1	1	0	1	1	1	1	1	0	0	1	1	1	0
CY046	1	9	1	150	40	1	1	1	1	0	0	1	1	0	1	1	1	1	0
CY047	2	8	1	137	32.5	1	1	1	0	1	0	0	1	1	0	0	1	1	0
CY048	2	9	1	132.5	39	1	1	1	1	1	0	1	1	1	0	1	1	1	0
CY049	2	9	1	133.5	24.5	1	1	0	1	0	1	1	1	0	0	0	1	1	0
CY050	1	9	1	134	31	1	1	0	1	1	1	1	0	1	0	1	1	1	0
CY051	1	6	1	118	26	1	1	0	1	1	0	1	0	0	0	1	1	1	0
CY052	1	9	1	139	36	0	1	0	1	1	0	0	1	0	1	1	1	1	0
CY053	2	7	1	134.5	29.5	1	0	0	1	0	1	0	0	0	0	0	1	0	0
CY054	2	6	1	122	24.5	1	0	0	1	1	1	1	1	1	1	1	1	1	0
CY055	1	9	1	138	29	1	1	1	0	1	0	0	0	1	0	1	0	0	0
CY056	2	9	1	135.5	28.5	1	1	0	1	1	1	0	1	0	0	0	1	1	0
CY057	1	8	1	130.5	21.5	1	1	0	0	1	1	1	1	0	0	1	1	1	1
CY058	2	7	1	132.5	37.5	1	1	0	1	0	1	0	0	0	1	0	1	1	0
CY059	1	8	1	132.5	30	1	1	1	1	0	1	1	1	0	0	1	1	1	0
CY060	2	6	1	120	23	1	1	0	0	1	1	1	1	1	0	0	1	1	0
CY061	1	7	1	132.5	29.5	1	1	0	0	1	1	1	1	0	0	1	1	0	0
CY063	2	7	1	127.5	28.5	1	1	1	1	0	1	0	0	1	0	1	1	1	0
CY064	1	7	1	131.5	27.5	1	1	0	1	1	1	1	1	1	1	1	1	1	1
CY065	2	6	1	117	21.5	1	1	1	1	0	1	1	1	0	1	0	1	0	0
CY066	2	6	1	122	23.5	1	1	0	0	0	0	1	1	1	1	1	1	1	0
CY067	1	9	1	130	31	1	1	1	1	0	1	1	1	1	0	1	1	1	0

CY068	1	8	1	125.5	25	1	0	0	1	0	1	1	1	1	1	1	1	0	0
CY069	1	9	1	134	34.5	1	1	0	0	0	1	0	0	0	0	1	1	1	1
CY070	2	9	1	138.5	36	1	1	0	1	0	1	1	1	1	1	1	1	1	1
CY071	2	8	1	129.5	31.5	1	1	0	0	1	0	0	0	1	1	1	1	0	0
CY072	1	8	1	137	35	1	1	0	1	0	0	0	0	0	1	1	1	1	1
CY073	1	6	1	122.5	25	1	1	0	0	0	0	1	0	1	1	1	1	0	1
CY074	1	8	1	128	26	1	1	0	0	1	1	0	1	0	0	0	1	0	0
CY075	1	8	1	125	20.5	1	1	0	0	1	1	1	0	0	0	0	0	0	0
CY076	1	7	1	131.5	32	1	1	0	1	0	1	0	1	0	0	1	1	0	1
CY077	2	7	1	126	25.5	1	1	0	0	0	0	0	0	0	0	0	0	0	0
CY078	1	7	1	120	24	1	1	0	0	1	1	1	0	0	0	1	0	1	0
CY079	1	6	1	114.5	19	1	1	0	0	0	1	0	0	0	0	1	1	0	0
CY080	1	7	1	130.5	28.5	1	1	0	0	0	1	0	0	0	0	0	1	0	0
CY081	2	7	1	123	27.5	1	1	0	1	1	0	1	1	0	0	0	1	0	1
CY082	2	6	1	118.5	22.5	1	1	0	1	0	1	0	1	0	0	1	0	0	0
CY083	1	6	1	122.5	22	1	1	0	0	0	1	0	1	0	0	1	0	0	0
CY084	2	9	1	135.5	32.5	1	0	0	0	0	0	0	1	0	0	0	1	0	1
CY085	2	7	1	124	27.5	1	1	1	0	0	1	1	0	0	0	1	1	1	0
CY086	2	9	1	136.5	32	1	1	0	1	1	0	0	0	0	0	1	1	1	0
CY087	1	9	1	136.5	32	1	1	0	1	0	1	0	0	0	0	1	1	1	0
CY088	1	8	1	128	24	1	1	0	0	0	1	1	1	0	1	1	1	1	0
CY089	2	7	1	125	24.5	1	1	0	0	1	1	1	1	0	1	1	1	0	0
CY090	1	6	1	113.5	21	1	1	0	0	1	1	1	1	0	0	1	0	0	0
CY091	2	7	1	125	27.5	0	1	0	1	1	1	0	1	0	0	1	1	1	1
CY092	1	9	1	134.5	33	1	1	0	1	0	0	1	1	0	0	1	1	0	0
CY093	1	9	1	138	34	1	1	0	1	0	0	1	0	0	0	1	0	1	1
CY094	1	7	1	132.5	28.5	1	1	0	1	0	1	1	0	1	0	1	1	0	0
CY095	1	8	1	127.5	26	1	1	1	0	1	1	0	0	0	0	1	1	0	1
CY096	1	9	1	137	32	1	1	0	1	0	1	0	1	0	0	1	0	0	0
CY097	2	6	1	120	27.5	1	1	0	1	0	1	0	1	0	0	0	1	0	0
CY098	1	7	1	129.5	26	1	1	1	1	0	0	1	0	1	1	1	1	0	0
CY099	2	7	1	126	27.3	1	1	0	1	1	1	1	1	0	0	1	1	0	0
CY100	2	6	1	106	20	1	1	0	1	1	1	0	1	0	0	0	1	1	0
CY101	1	7	1	131.5	31.5	1	1	0	0	1	1	0	1	0	1	0	1	1	1
CY102	2	9	1	133.5	27	0	1	0	0	0	1	1	1	0	0	0	1	1	1
CY103	2	6	1	119	28	1	1	0	0	0	1	1	0	1	0	1	1	1	1
CY104	1	9	1	131	25	1	1	0	0	1	1	1	1	0	0	1	1	1	1
CY105	2	9	1	135.5	27	1	1	0	1	0	1	1	0	1	0	1	1	0	1
CY106	1	6	1	122.5	24	1	1	0	1	1	0	0	1	0	0	0	1	0	1
CY107	2	8	1	127	20	1	1	0	0	1	0	0	0	0	0	1	1	0	0
CY108	1	6	1	120.5	20	1	1	1	1	0	0	1	0	0	0	1	1	0	1
CY109	2	7	1	127	26.5	1	1	0	1	1	1	1	1	1	0	1	1	0	1
CY110	2	9	1	129.5	26	1	1	1	1	0	1	1	1	1	0	1	1	1	1
CY111	2	6	1	122	22	1	1	1	0	1	1	1	1	1	1	1	1	1	1
CY112	1	6	1	122	23.5	1	1	0	0	0	1	1	1	0	1	1	1	0	1
CY113	1	8	1	134	26	1	1	0	1	0	1	1	1	1	0	1	1	0	0
CY114	1	8	1	132	25.5	1	1	0	0	0	1	0	1	0	0	1	1	1	0
CY115	2	6	1	119	21.5	1	1	0	0	0	1	1	1	0	0	0	1	0	1
CY116	1	9	1	135	28	1	1	0	1	0	1	1	1	1	0	1	1	1	1
CY117	2	7	1	123.5	24.5	1	1	0	1	1	1	0	1	1	0	1	1	1	0
CY118	1	8	1	130	25	1	1	0	1	0	1	1	1	1	1	1	1	1	1
CY119	2	8	1	136.5	33.5	1	1	0	0	1	0	0	0	0	0	1	0	1	1
CY120	1	9	1	131	31	1	1	0	0	1	1	1	0	0	0	0	0	0	0
CY121	2	9	1	133.5	29.5	1	1	0	0	1	1	1	1	0	1	1	1	1	1
CY122	2	7	1	127.5	23.5	1	1	0	0	0	1	0	0	0	0	0	1	0	0
CY123	2	7	1	125.5	21	1	1	0	0	1	1	0	1	0	0	0	1	0	0
CY124	2	7	1	126	22	1	1	1	0	1	1	1	1	1	1	0	1	1	1
CY125	2	6	1	115	18	1	1	0	0	0	0	0	0	0	0	1	0	0	0
CY126	1	8	1	136	28.5	1	1	1	1	1	1	1	1	0	1	1	1	1	0
CY127	2	8	1	129.5	30	1	1	1	1	0	1	1	1	1	0	1	1	1	0
CY128	1	9	1	130	29	1	1	1	1	1	1	0	1	1	1	1	1	1	1
CY129	1	9	1	148	30	1	1	1	1	1	1	1	1	0	1	1	1	1	0
CY130	2	8	1	133	32.5	1	0	0	1	0	1	1	0	1	0	1	1	0	1
CY131	2	8	1	132	28	1	1	0	0	1	1	1	1	0	0	1	0	0	0
CY132	1	9	1	147	46	1	1	1	1	1	1	1	1	1	1	1	1	1	1
CY133	1	7	1	125.5	27	1	1	1	1	1	1	1	1	1	1	1	1	1	1
CY134	2	8	1	127	22.5	1	1	1	1	1	1	1	0	0	0	1	0	0	0
CY135	1	7	1	125.5	21.5	1	1	0	0	0	1	0	0	0	0	0	1	0	0
CY136	2	9	1	128	30.5	1	1	1	1	1	1	0	1	1	1	1	1	1	1

CY137	1	9	1	141	41	1	1	0	0	0	1	0	0	0	0	1	0	0	0
CY138	1	6	1	126.5	27	1	1	1	1	0	0	1	1	0	0	0	1	1	0
CY139	1	6	1	115	21	0	0	0	0	0	0	0	0	0	0	0	0	0	0
CY140	1	7	1	125.5	24	1	0	0	1	1	0	0	0	0	0	1	0	0	0
CY141	1	6	1	119	23	1	1	1	1	1	1	1	1	1	1	1	1	0	1
CY142	1	7	1	118.5	26	1	1	1	1	1	1	0	1	1	1	1	1	1	1
CY143	1	7	1	129.5	30	1	1	1	1	1	1	1	1	1	1	1	1	1	1
CY144	1	9	1	137	37.5	1	1	0	0	1	1	1	1	0	0	1	1	1	0
CY145	2	6	1	114	17.5	0	0	0	0	0	0	0	0	0	0	0	0	0	0
CY146	2	8	1	121.5	23.5	1	1	0	1	0	1	1	1	1	0	1	1	1	0
CY147	1	8	1	128	26	1	1	0	1	1	1	1	1	0	0	1	1	1	1
CY148	2	9	1	134.5	33.5	1	0	0	1	0	1	0	1	0	0	0	1	0	0
CY149	1	8	1	135.5	30.5	1	1	0	1	0	0	1	0	0	0	1	1	0	0
CY150	1	7	1	129.5	27	1	1	0	1	1	0	0	0	0	0	1	1	1	0
CY151	1	7	1	129.5	33	1	0	0	0	0	1	0	0	1	0	1	0	0	0
CY152	2	7	1	123	25.5	0	1	0	0	1	0	1	0	0	0	0	1	1	0
CY153	1	7	1	122	23.5	1	1	1	0	0	1	1	0	1	1	1	1	1	1
CY154	1	7	1	125.5	23	1	1	0	0	0	0	0	1	0	0	1	1	1	0
CY155	1	7	1	131.5	31	0	1	0	1	0	0	1	0	0	0	0	1	1	1
CY156	1	7	1	123	26	1	1	0	1	0	1	0	0	0	1	1	1	1	0
CY157	2	6	1	117.5	21.5	0	1	0	1	0	0	1	0	0	0	0	1	1	1
CY158	1	7	1	125.5	25	1	1	0	1	0	0	1	0	0	0	0	0	0	0
CY159	1	7	1	125.5	26	1	0	0	1	0	1	0	0	0	0	0	1	0	0
CY160	1	9	1	143	26	1	1	1	1	1	1	1	1	1	1	1	1	1	0
CY161	1	7	1	122.5	20	1	1	1	1	1	1	0	1	1	0	1	1	1	1
CY162	1	9	1	132.5	32	1	0	0	0	0	0	0	0	0	0	0	0	0	0
CY163	1	7	1	124.5	21.5	1	1	0	0	0	0	0	0	0	0	0	0	0	0
CY164	2	7	1	123.5	21.5	0	1	0	0	0	1	0	0	0	0	1	0	1	0
CY165	2	7	1	118	20.5	1	1	0	0	1	0	0	0	0	0	1	1	0	0
CY166	1	6	1	120.5	22	1	1	0	0	0	0	0	0	0	0	0	0	0	0
CY167	2	8	1	123.5	20	1	1	0	1	0	1	1	1	1	0	1	1	1	1
CY168	2	8	1	127	25	1	1	1	1	1	1	1	1	0	1	1	1	1	0
CY169	1	7	1	131	34	1	1	0	0	0	1	1	1	0	0	0	1	0	1
CY170	1	9	1	137.5	28	1	1	0	1	0	1	1	1	1	0	1	1	1	1
CY171	1	8	1	137.5	40.5	1	1	0	1	1	0	0	0	0	0	1	1	1	0
CY172	1	7	1	129	32.5	1	1	0	1	0	0	1	0	0	0	1	1	0	0
CY173	2	6	1	117.5	22	1	1	0	1	0	1	1	1	1	0	1	0	0	0
CY174	1	9	1	139	41.5	1	1	0	0	0	1	0	0	0	0	0	1	0	0
CY175	2	9	1	126.5	31	1	0	0	0	0	0	0	1	0	0	0	1	0	1
CY176	1	6	1	121.5	21.5	1	0	0	1	0	1	0	0	0	0	0	1	0	0
CY177	2	8	1	125	25.5	1	1	0	1	1	1	1	1	1	0	1	1	1	1
CY178	1	7	1	122	26	1	1	0	1	1	1	1	1	1	1	1	1	1	1
CY179	2	7	1	127	26.5	1	1	0	0	0	0	0	0	1	0	1	1	0	0
CY180	2	8	1	130	29.5	0	1	0	0	1	0	1	0	0	0	1	1	1	0
CY181	1	9	1	132	24	1	0	0	0	0	0	0	0	0	0	1	1	1	0
CY182	1	7	1	121.5	20	1	0	0	1	0	0	0	0	0	0	0	0	0	0
CY183	2	7	1	119.5	23.5	1	1	1	1	1	1	1	1	1	0	1	1	1	1
CY184	2	8	1	132.5	36	1	1	0	0	0	1	1	1	0	0	0	1	0	1
CY185	1	9	1	136.5	32.5	1	0	0	0	0	1	0	1	1	0	1	0	0	0
CY186	2	7	1	126.5	25.5	0	1	1	1	0	0	0	0	0	0	1	1	1	0
CY187	2	8	1	143	41.5	1	1	0	0	0	0	0	0	0	0	0	0	0	0
CY188	2	8	1	125.5	20	1	1	0	1	0	1	1	1	1	1	1	1	1	1
CY189	1	7	1	123.5	23	1	1	0	0	0	1	0	1	0	0	1	0	0	0
CY190	1	6	1	119.5	23	1	1	0	1	1	0	0	1	0	0	0	1	0	1
CY191	1	9	1	139	32	1	1	0	0	1	1	0	1	0	0	0	1	0	0
CY192	2	7	1	120.5	20.5	1	1	0	0	0	0	0	0	0	0	0	0	0	0
CY193	2	7	1	128.5	23.5	0	0	0	0	0	0	0	0	0	0	0	0	0	0
KM001	2	7	2	119.5	22	1	1	0	0	0	1	1	1	0	0	1	0	1	1
KM002	1	6	2	118	21	0	1	0	0	0	0	0	0	0	0	0	0	0	0
KM003	2	8	2	127	25	1	1	0	1	0	1	0	1	0	0	0	1	1	0
KM004	1	6	2	116	21	1	1	0	0	1	0	0	1	1	0	1	0	1	1
KM005	2	8	2	133.5	26	1	1	0	1	0	1	0	1	0	0	0	1	1	0
KM006	2	7	2	124.5	25	1	1	0	0	0	0	0	1	0	0	1	0	0	0
KM007	2	9	2	133	31	1	0	0	1	0	1	0	1	0	0	0	1	0	0
KM008	1	6	2	127.5	31	1	1	0	0	0	0	0	1	0	0	1	1	1	0
KM009	1	9	2	131.5	29.5	1	1	1	1	1	1	1	1	0	1	1	1	1	1
KM010	1	8	2	130	31.5	1	1	1	1	1	0	1	1	1	0	1	1	1	0
KM011	1	9	2	135.5	31.5	1	1	0	1	1	1	0	1	0	0	1	1	1	1
KM012	2	7	2	125.5	24.5	1	1	1	1	0	0	1	1	0	0	0	1	1	1

KM013	2	8	2	129.5	32	1	1	0	1	0	1	1	1	0	0	0	1	1	0
KM014	2	8	2	135.5	36.5	1	1	1	1	1	0	1	1	1	0	1	1	1	0
KM015	1	9	2	138	30	1	1	1	1	1	1	1	1	1	1	1	1	1	0
KM016	2	9	2	134	41	1	1	1	1	1	1	1	1	1	1	1	1	1	0
KM017	1	9	2	132	34	1	1	0	0	1	1	0	1	0	0	0	1	0	0
KM018	2	6	2	119.5	25.5	1	0	0	1	0	1	0	0	0	0	0	0	0	0
KM019	1	8	2	135	32	1	1	1	1	1	1	0	1	1	1	1	0	0	1
KM020	2	8	2	127	22	1	1	0	0	1	1	0	1	0	1	1	0	0	1
KM021	2	8	2	130.5	23.5	1	0	0	0	0	0	0	0	0	0	1	1	1	0
KM022	2	6	2	117.5	19	1	0	0	1	0	0	0	0	0	0	1	0	0	0
KM023	1	9	2	133	25	1	1	1	0	1	1	0	0	0	0	1	1	1	0
KM024	2	8	2	137	28.5	1	0	0	0	0	0	0	0	0	0	0	1	0	1
KM025	1	7	2	128.5	29.5	1	1	0	1	0	1	0	1	0	0	1	1	1	1
KM026	1	8	2	139	36.8	1	1	0	1	0	0	1	1	0	0	1	1	0	0
KM027	1	6	2	117	21	1	1	0	1	0	1	0	0	0	0	1	1	1	0
KM028	1	8	2	129	28	1	1	1	1	1	1	1	1	0	1	1	1	1	0
KM029	1	8	2	139.5	37.5	1	1	0	0	1	0	1	1	1	1	0	0	0	0
KM030	1	8	2	131	29.5	1	1	0	1	0	1	1	0	0	1	1	1	0	1
KM031	1	8	2	140	32	1	1	0	1	0	0	1	0	0	0	1	0	1	1
KM032	2	7	2	125.5	21.5	1	1	1	1	1	1	0	1	0	0	0	1	0	1
KM033	2	6	2	119.5	23.5	1	1	0	1	1	1	1	1	1	0	1	1	1	1
KM034	1	8	2	127.5	23	1	0	0	0	0	0	0	0	0	0	0	0	0	0
KM035	1	8	2	127	25.5	1	1	0	1	1	1	1	1	1	0	1	1	1	1
KM036	2	6	2	115.5	18	1	1	0	0	1	0	0	0	0	0	1	0	1	1
KM037	2	6	2	128	33	1	1	0	1	1	0	1	1	0	1	1	1	1	1
KM038	1	7	2	127.5	31.5	1	1	0	1	1	0	1	0	0	0	1	0	0	0
KM039	2	6	2	114	19	0	0	0	0	0	0	0	0	0	0	0	0	0	0
KM040	2	7	2	125.5	22	1	1	0	0	1	1	1	1	0	0	1	1	1	0
KM041	2	8	2	127	22	0	1	0	0	1	0	1	0	0	0	0	1	1	0
KM042	1	6	2	120.5	22	1	1	0	0	0	0	0	0	0	0	0	0	0	0
KM043	2	7	2	125.5	24	0	1	0	0	0	1	1	1	0	0	0	1	1	1
KM044	2	8	2	136	30	1	1	0	0	1	1	1	0	0	0	0	0	0	0
KM045	2	8	2	132.5	26.5	0	0	0	0	0	0	0	0	0	0	0	0	1	0
KM046	1	7	2	123.5	21.5	1	1	0	1	0	1	0	1	0	1	1	1	0	1
KM047	2	6	2	115.5	20	1	0	0	1	1	0	0	0	0	0	1	0	0	0
KM048	2	8	2	127	22	1	1	0	0	1	1	0	1	0	0	1	0	0	1
KM049	2	8	2	123	23.5	1	1	0	1	1	0	0	0	0	0	1	1	1	0
KM050	1	6	2	121	23.5	1	1	0	1	0	1	0	1	0	0	1	1	1	0
KM051	2	9	2	136.5	32	1	1	0	0	1	1	0	0	0	0	0	1	0	0
KM052	2	6	2	119.5	20.5	0	1	0	0	0	0	0	0	0	0	0	0	0	0
KM053	2	8	2	133	22	1	1	0	1	1	1	1	1	0	0	1	1	1	1
KM054	2	9	2	146	28	1	1	1	1	1	1	0	1	1	0	1	0	1	1
KM055	2	6	2	122	22	1	1	0	1	1	1	1	0	0	0	1	1	1	0
KM056	2	6	2	112	18	1	1	0	0	0	1	0	0	0	0	0	1	0	0
KM057	2	9	2	126	26	1	1	1	1	1	1	1	1	0	1	0	1	1	1
KM058	1	6	2	121	24.5	1	1	0	0	0	0	0	1	0	0	1	1	0	0
KM059	1	6	2	120.5	27	1	1	0	1	0	1	1	1	1	1	1	1	1	0
KM060	2	7	2	128.5	24.5	1	1	0	1	1	1	1	1	1	1	0	1	1	0
KM061	1	8	2	125.5	22	0	1	0	0	1	0	1	0	0	0	0	1	1	0
KM062	2	6	2	117	18	1	1	0	1	0	0	1	0	0	0	0	0	0	0
KM063	1	6	2	117	23	1	1	0	1	0	1	0	1	0	0	0	1	1	0
KM064	1	7	2	122	24.5	1	1	1	1	0	1	1	1	1	0	1	1	1	1
KM065	1	6	2	121.5	20.5	0	0	0	0	1	1	0	0	0	0	1	0	1	0
KM066	1	6	2	123	25	0	0	0	0	0	0	0	0	0	0	0	0	0	0
KM067	1	9	2	140	37.5	1	1	1	1	1	1	1	1	1	1	1	1	1	1
KM068	1	8	2	132	28.5	1	1	1	1	1	1	1	1	1	1	1	1	1	0
KM069	1	7	2	128.5	39	1	1	0	0	0	0	0	1	1	0	0	1	0	1
KM070	1	8	2	130.5	30	1	1	0	1	0	1	1	1	1	1	1	1	1	0
KM071	1	9	2	133	23.5	1	1	1	0	1	1	1	1	1	1	1	1	1	1
KM072	2	9	2	135	32.5	1	1	1	1	1	1	1	1	0	0	1	1	0	0
KM073	2	6	2	112	17.5	1	1	0	1	1	1	1	1	1	1	1	1	1	0
KM074	1	8	2	128	28	1	0	0	1	0	1	0	0	0	0	0	0	0	0
KM075	2	7	2	123.5	24	1	0	0	0	0	0	0	0	0	0	0	0	0	0
KM076	2	6	2	118.5	26	1	1	0	0	1	0	0	1	1	1	1	1	1	0
KM077	2	6	2	122	28	1	1	0	0	0	0	0	0	0	0	0	0	0	0
KM078	2	6	2	117	21	1	1	0	0	0	1	0	0	1	1	1	1	1	1
KM079	1	7	2	127.5	29	0	1	0	0	0	0	0	0	0	0	0	0	0	0
KM080	1	7	2	132.5	26	1	1	0	0	0	1	0	0	0	0	1	0	0	0
KM081	2	6	2	124	24.5	1	1	1	1	0	1	1	1	1	1	1	1	1	0

KM082	2	6	2	109.5	17	1	1	0	1	0	1	1	1	1	1	1	1	1	1
KM083	2	6	2	118	24.5	0	1	0	0	0	1	0	0	0	0	1	0	0	0
KM084	2	6	2	118	23	1	1	1	1	0	1	0	1	1	0	1	1	1	1
KM085	2	9	2	134	29.5	1	1	0	1	1	1	1	1	0	0	1	1	0	1
KM086	1	8	2	129	30.5	1	1	0	0	1	1	0	0	0	0	0	1	0	0
KM087	1	9	2	138	35	1	1	0	1	0	1	0	1	0	0	0	1	0	0
KM088	2	6	2	119.5	23	1	0	0	0	0	0	0	0	0	0	0	0	0	0
KM089	1	7	2	125.5	23.5	0	0	0	0	0	0	0	0	0	0	0	0	0	0
KM090	2	6	2	113	18	1	0	0	0	0	0	0	0	0	0	0	0	0	0
KM091	2	8	2	128.5	26	1	0	0	1	0	1	0	0	0	0	0	0	0	0
KM092	1	9	2	135.5	31.5	1	1	0	1	1	1	1	1	0	1	1	1	1	0
KM093	2	9	2	128.5	28.5	1	1	1	1	0	1	1	1	1	1	1	0	1	1
KM094	1	9	2	133	30.5	1	1	0	1	1	1	1	1	0	0	1	1	1	0
KM095	2	7	2	124.5	21.5	0	0	0	0	1	1	0	0	0	0	1	0	1	0
KM096	1	9	2	141.5	32	1	1	0	1	0	0	1	1	0	0	1	0	1	0
KM097	2	9	2	136	37	1	1	1	1	1	1	1	1	0	1	1	1	1	1
KM098	1	8	2	127	22	1	1	0	1	1	0	1	0	0	0	1	0	0	0
KM099	1	9	2	133	32	1	1	1	1	1	1	1	1	1	1	1	1	1	1
KM100	2	9	2	138	35.5	1	1	0	1	1	0	1	1	0	1	1	1	0	1
KM101	1	8	2	127	27	1	1	1	1	0	1	0	1	0	1	1	1	1	0
KM102	2	7	2	124.5	34.5	1	1	0	0	0	0	0	0	1	1	1	1	0	0
KM103	2	8	2	130	28.5	1	1	0	0	1	0	0	1	0	0	1	1	1	0
KM104	2	7	2	125.5	24.5	1	1	1	1	1	1	1	1	1	1	1	1	1	0
KM105	2	7	2	129.5	27	1	1	0	0	0	0	0	0	0	0	0	0	0	0
KM106	1	7	2	127.5	24.5	1	0	0	1	0	1	0	1	0	0	1	1	1	1
KM107	1	7	2	128.5	26	0	1	0	0	1	0	1	0	0	0	1	1	1	0
KM108	2	8	2	133	24	1	1	0	0	1	1	0	0	1	0	0	0	1	1
KM109	2	8	2	127.5	28	1	1	0	0	0	1	0	0	0	0	1	1	0	0
KM110	1	8	2	127.5	26	1	1	1	1	1	1	0	1	1	0	1	1	1	1
KM111	1	9	2	135.5	29	1	1	1	1	1	1	1	1	1	1	1	1	1	1
KM112	1	9	2	134.5	35	1	1	0	1	1	1	1	1	0	1	1	1	1	1
KM113	2	6	2	120	32	0	0	0	0	0	0	0	0	0	0	0	0	0	0
KM114	1	7	2	123.5	23.5	1	1	0	1	1	0	0	1	0	0	1	1	0	0
KM115	2	6	2	111.5	19	1	1	0	1	1	1	1	0	0	0	1	1	0	1
KM116	1	7	2	125.5	24	0	0	0	0	0	0	0	0	0	0	0	0	0	0
KM117	2	6	2	118	22	1	1	0	0	0	0	0	0	0	0	0	0	0	0
KM118	2	7	2	116.5	19	1	1	1	1	0	1	1	1	1	0	1	1	1	1
KM119	2	7	2	126.5	35.5	1	1	1	0	0	1	1	0	0	0	0	0	1	1
KM120	1	8	2	128	26.5	1	1	1	1	1	1	1	1	1	1	1	1	1	0
KM121	1	7	2	123	27	1	1	0	0	1	1	0	0	1	0	1	1	1	0
KM122	1	6	2	121.5	24	1	1	0	0	0	0	0	0	0	0	1	0	0	0
KM123	2	9	2	134	28	1	0	0	0	0	0	1	0	0	0	1	0	0	0
KM124	2	6	2	122	20.5	1	1	0	0	0	1	1	1	0	0	1	1	0	0
KM125	2	9	2	139.5	33	1	1	0	0	0	1	0	1	0	0	1	1	1	0
KM126	1	6	2	121.5	25	1	1	0	1	0	1	0	0	1	0	0	1	1	0
KM127	1	6	2	125.5	22	1	1	0	0	0	0	0	0	1	0	0	1	0	1
KM128	1	6	2	117	22	1	1	1	1	1	1	1	1	0	1	1	1	1	1
KM129	1	8	2	135	25.5	1	1	1	1	1	1	1	1	1	1	1	1	1	1
KM130	2	6	2	120	19	0	0	0	0	0	0	0	0	0	0	0	0	0	0
KM131	2	8	2	130	31	1	1	0	0	1	0	1	1	1	0	0	0	0	0
KM132	2	8	2	137.5	30	1	0	0	1	1	0	0	0	0	0	0	0	0	0
KM133	1	8	2	129	25.5	1	1	1	1	1	1	1	1	1	1	1	1	1	1
KM134	1	6	2	126.5	27	1	1	0	0	0	1	0	0	0	0	1	1	1	1
KM135	2	6	2	117	21	1	1	0	0	1	1	0	0	0	1	0	1	0	0
KM136	2	7	2	126.5	25.5	1	1	0	1	1	1	1	0	1	0	1	1	1	1
KM137	1	8	2	135.5	31.5	1	1	0	1	1	0	0	1	0	0	0	1	0	1
KM138	1	6	2	120.5	23.5	1	1	0	0	0	1	1	1	0	0	1	1	0	0
KM139	1	8	2	132.5	30.5	1	1	0	0	1	1	1	0	0	0	1	1	1	0
KM140	2	7	2	125	25	1	0	0	0	0	1	0	1	1	1	1	0	0	0
KM141	1	9	2	140.5	37	1	1	0	1	1	0	0	0	0	0	1	1	1	0
KM142	2	9	2	132.5	23	1	1	0	1	1	1	1	1	1	1	1	1	1	0
KM143	2	6	2	127	23.5	1	0	0	1	0	1	0	0	0	0	0	0	0	0
KM144	2	6	2	120	19	1	1	1	0	0	1	1	0	0	0	0	0	1	1
KM145	2	7	2	125	25	1	1	0	1	1	0	1	0	0	0	1	0	0	0
KM146	1	8	2	133	35.5	1	1	0	0	0	0	0	1	0	0	1	1	0	0
KM147	1	9	2	139	45	1	1	0	1	1	1	0	1	0	0	0	1	0	0
KM148	1	6	2	121.5	21.5	1	1	0	0	0	1	1	1	0	0	1	1	0	0
KM149	1	7	2	131.5	30.5	1	1	0	0	1	1	1	0	0	0	0	0	0	0
KM150	2	8	2	127	27.5	1	1	0	1	1	1	1	1	1	1	1	1	1	1

KM151	1	7	2	126.5	27.5	1	1	0	1	1	0	1	0	0	0	1	0	0	0
KM152	1	7	2	126	27.5	1	1	0	1	1	1	1	1	1	1	1	1	1	0
KM153	2	7	2	123.5	23	1	1	0	1	0	1	1	1	1	0	1	1	1	1
KM154	2	9	2	147.5	38.5	1	1	0	1	1	1	0	0	0	0	1	1	1	1
KM155	1	7	2	118.5	22.5	1	1	0	0	1	1	0	1	0	0	1	1	1	0
KM156	1	7	2	130.5	31	1	1	0	0	0	0	0	1	1	0	0	1	0	1
KM157	2	6	2	113	17	1	0	0	0	0	0	0	0	0	0	0	0	0	0
KM158	1	8	2	128.5	24.5	0	1	0	0	0	0	0	0	0	0	0	0	0	0
KM159	1	6	2	117	24	0	0	0	0	0	0	0	0	0	0	0	0	1	0
KM160	2	6	2	120	25	0	0	0	0	0	0	0	0	0	0	0	0	0	0
KM161	2	9	2	140	40	1	1	0	1	0	1	0	0	0	0	1	1	1	0
KM162	2	6	2	113	21	0	0	0	0	0	0	0	0	0	0	0	0	0	0
KM163	2	9	2	129.5	28.5	1	1	0	1	1	0	0	1	0	0	1	1	0	0
KM164	1	9	2	138	32	1	1	0	0	0	0	0	0	1	0	1	1	0	0
KM165	1	9	2	137.5	31	1	1	1	1	1	1	1	1	1	1	1	1	1	0
KM166	1	8	2	133.5	30	1	1	0	1	1	1	0	1	0	0	1	0	0	0
KM167	2	6	2	121	23.5	1	1	0	0	0	0	0	0	0	0	1	0	0	0
KM168	1	7	2	128.5	26	1	1	0	0	1	1	0	1	0	0	1	1	0	1
KM169	1	7	2	131.5	30.5	1	1	0	0	0	0	0	0	0	0	1	0	0	0
KM170	1	7	2	130.5	26.5	1	1	1	0	0	0	1	0	0	0	1	1	1	1
KM171	2	6	2	120	19.5	1	1	0	1	0	1	0	1	1	1	1	1	1	1
SN001	1	9	3	139	38	1	1	0	1	1	1	0	1	0	0	1	1	1	1
SN002	2	7	3	121.5	21	1	1	0	1	1	1	1	1	0	0	1	1	1	1
SN003	2	8	3	133	27.5	1	1	0	1	1	1	0	1	0	0	1	1	1	0
SN004	1	9	3	141.5	40	1	1	0	1	1	1	0	1	0	1	1	1	1	0
SN005	2	8	3	128	20	1	1	0	1	0	0	1	0	0	0	1	1	0	0
SN006	1	8	3	132	31	1	1	0	1	1	0	1	1	0	0	1	1	0	0
SN007	2	8	3	131	27	1	1	0	0	1	0	0	0	0	0	1	0	1	1
SN008	2	8	3	131	28.5	1	1	0	1	1	1	0	1	0	1	1	1	0	0
SN009	2	6	3	115.5	19.5	1	1	0	1	1	1	0	0	0	1	1	1	1	0
SN010	1	9	3	128.5	31	1	1	0	1	0	1	1	1	0	0	1	1	0	1
SN011	1	8	3	139.5	41	1	1	0	1	0	1	0	1	0	0	1	1	1	1
SN012	1	8	3	133.5	31	1	1	0	1	1	1	0	1	0	0	1	1	1	0
SN013	1	6	3	122.5	25.5	1	1	0	1	1	1	0	0	0	0	1	1	1	0
SN014	1	6	3	113.5	21	1	1	0	1	1	1	1	0	0	1	1	0	0	0
SN015	2	7	3	123.5	23	1	1	0	1	1	1	0	1	0	0	1	0	1	0
SN016	1	9	3	136.5	37	1	1	0	1	1	0	0	0	0	0	1	1	1	0
SN017	2	6	3	114.5	18	1	1	0	1	1	0	0	1	0	0	1	1	0	1
SN018	1	9	3	144.5	39	1	1	0	1	1	0	1	1	0	1	1	1	0	0
SN019	2	8	3	136.5	31	1	1	0	1	1	1	1	0	0	0	1	1	0	0
SN020	1	8	3	129	31	1	1	0	1	1	1	1	1	0	0	1	0	0	0
SN021	2	7	3	123.5	22.5	1	1	0	1	1	1	1	1	1	0	1	1	1	0
SN022	2	7	3	123.5	24	1	1	0	1	1	0	1	1	0	0	1	1	1	1
SN023	1	6	3	120	22	1	1	0	1	0	1	1	1	0	1	1	1	0	0
SN024	2	6	3	112	17	1	1	0	1	0	1	1	1	0	0	1	1	1	0
SN025	2	6	3	115	17	1	1	0	1	1	1	0	1	0	1	1	1	1	1
SN026	2	8	3	130	33	1	1	0	1	1	1	1	0	0	0	1	1	0	1
SN027	2	8	3	130.5	33	1	1	0	1	1	1	0	1	0	0	1	1	0	0
SN028	1	7	3	125	26.5	1	1	0	1	1	1	1	1	0	0	1	1	1	1
SN029	2	8	3	127	27.5	1	1	0	1	1	0	1	1	0	0	1	1	0	0
SN030	2	6	3	114	17.5	1	1	0	1	0	0	1	1	0	0	1	1	0	1
SN031	1	6	3	116	18	1	1	0	1	0	1	1	1	1	1	1	1	1	0
SN032	2	7	3	120.5	16.5	1	1	0	1	1	1	1	1	0	0	1	1	0	0
SN033	2	8	3	127.5	26.5	1	1	0	1	1	0	1	0	1	0	1	1	1	1
SN034	1	7	3	121.5	21.5	1	1	0	1	1	1	0	0	0	0	1	1	1	1
SN035	2	6	3	112.5	17.5	1	1	0	1	1	1	0	1	0	0	1	1	0	0
SN036	1	7	3	120.5	22.5	1	1	0	1	1	1	1	1	0	0	1	1	0	0
SN037	1	6	3	117.5	26	1	1	0	1	1	1	1	0	0	1	1	1	1	1
SN038	1	8	3	125.5	24	1	1	0	1	1	1	1	1	0	0	1	1	1	0
SN039	2	6	3	112.5	18.5	1	1	0	1	1	1	1	1	0	0	1	1	0	0
SN040	1	9	3	144.5	37	1	1	0	1	1	1	0	1	0	1	1	1	1	0
SN041	1	6	3	112.5	18.5	1	1	0	1	0	1	0	1	0	1	1	1	0	0
SN042	2	9	3	138	32	1	1	1	1	1	1	1	1	0	0	1	1	0	0
SN043	1	9	3	144	36.5	1	1	0	1	1	1	1	1	0	0	1	1	1	0
SN044	2	8	3	130	27.5	1	1	0	1	0	1	1	0	0	1	1	1	0	0
SN045	2	6	3	120.5	18	1	1	0	1	1	0	1	1	0	1	1	1	1	0
SN046	1	6	3	115.5	24	1	1	0	1	1	0	1	1	0	0	1	1	1	0
SN047	1	9	3	141.5	26.5	1	1	0	1	1	1	0	1	0	1	1	1	1	0
SN048	1	8	3	123.5	24.5	1	1	0	1	1	1	0	1	0	1	0	1	0	0

SN049	2	7	3	123	26	1	1	0	0	1	1	0	1	0	0	1	1	0	0
SN050	2	8	3	124	23.5	1	1	0	1	1	1	1	1	0	0	1	1	0	0
SN051	1	7	3	130.5	24.5	1	1	0	1	1	1	1	0	0	0	1	1	1	0
SN052	2	9	3	134	30	1	1	0	1	1	1	1	1	0	0	1	1	1	0
SN053	2	6	3	111	17	1	1	0	1	1	1	0	1	0	0	1	1	1	0
SN054	1	6	3	121.5	20	1	1	0	1	1	1	1	1	0	1	1	0	1	0
SN055	1	9	3	135	29	1	1	0	1	1	1	1	0	0	0	1	0	0	0
SN056	2	9	3	144.5	38	1	1	0	1	0	1	1	1	1	0	1	1	1	0
SN057	1	7	3	129.5	29	1	1	0	1	1	1	0	1	0	0	1	1	1	1
SN058	1	9	3	145.5	41.5	1	1	0	1	1	1	0	0	0	1	1	1	1	0
SN059	2	6	3	122.5	27.5	1	1	0	1	1	1	1	0	0	0	1	1	1	0
SN060	2	9	3	142	38	1	1	0	1	1	1	1	0	0	0	1	1	1	1
SN061	1	8	3	128.5	31.5	1	1	0	1	1	0	1	1	1	0	1	0	0	0
SN062	1	7	3	124.5	23.5	1	1	0	1	1	0	1	1	0	0	1	1	0	0
SN063	1	7	3	129.5	29	1	1	0	1	0	1	1	1	0	1	1	1	0	1
SN064	1	6	3	116	18.5	1	1	0	1	1	1	1	1	0	0	1	1	1	0
SN065	1	8	3	132	35	1	1	0	1	1	1	0	1	0	0	1	1	1	0
SN066	1	6	3	110.5	20	1	1	0	0	1	1	0	1	0	1	1	1	0	0
SN067	2	6	3	110.5	15	1	1	0	0	1	1	0	1	1	0	1	1	0	0
SN068	2	9	3	150.5	44.5	1	1	0	1	1	1	1	1	0	0	1	1	1	1
SN069	2	6	3	119	21.5	1	1	0	1	0	1	1	1	0	0	1	1	0	0
SN070	1	9	3	137	42.5	1	1	0	1	1	0	1	1	1	0	1	1	1	0
SN071	1	7	3	129.5	29	1	1	0	0	1	1	1	1	0	0	1	1	1	1
SN072	1	7	3	128.5	31.5	1	1	0	0	1	1	0	1	0	0	1	1	0	1
SN073	2	6	3	108.5	14.5	1	1	0	1	0	1	0	1	0	0	1	1	0	1
SN074	1	9	3	130	29	1	1	0	0	1	1	0	1	0	0	1	0	0	1
SN075	2	6	3	120	22	1	1	0	1	1	0	1	1	0	0	1	0	0	1
SN076	2	7	3	125.5	22	1	1	0	1	1	0	1	1	0	1	1	1	1	0
SN077	1	9	3	142	42	1	1	0	1	1	0	1	0	0	0	1	0	0	0
SN078	2	9	3	146.5	44.5	1	0	0	1	1	0	1	1	0	1	1	0	1	0
SN079	2	9	3	132.5	34.5	1	1	1	1	1	1	1	1	0	0	1	0	0	0
SN080	1	8	3	134.5	41.5	1	1	0	1	1	1	0	1	0	1	1	0	0	0
SN081	1	6	3	121.5	20	1	1	0	1	1	0	0	1	0	0	1	1	0	0
SN082	2	6	3	117	21	1	0	0	1	1	0	0	0	0	0	1	0	0	0
SN083	2	8	3	126	23.5	1	1	0	1	1	1	1	0	0	0	1	0	1	0
SN084	1	6	3	112	17.5	1	1	0	1	1	1	1	1	0	1	1	1	1	1
SN085	1	6	3	118.5	22	1	1	0	1	1	1	1	1	0	0	1	0	1	0
SN086	1	8	3	129.5	31.5	1	0	0	1	1	1	0	1	0	0	1	1	1	1
SN087	2	6	3	121	20	1	1	0	1	1	1	0	1	0	1	1	0	1	0
SN088	1	6	3	115.5	20	1	1	0	1	1	1	0	0	0	0	1	1	0	0
SN089	2	9	3	145.5	52.5	1	1	0	1	1	1	1	1	0	0	1	1	1	0
SN090	1	6	3	119	25	1	1	0	1	1	1	1	1	0	0	1	0	0	1
SN091	1	8	3	127.5	30.5	1	0	0	0	0	1	0	1	1	1	1	0	0	0
SN092	2	8	3	127	25.5	1	1	0	0	1	1	1	1	0	0	1	0	1	1
SN093	1	7	3	124	29.5	1	1	0	1	0	1	0	1	1	1	1	0	0	0
SN094	2	6	3	121.5	24	1	1	0	0	1	1	0	1	0	1	1	1	0	0
SN095	2	6	3	117.5	21	1	1	0	1	1	1	1	1	0	0	1	0	1	0
SN096	1	9	3	139	32.7	1	1	0	1	1	1	1	1	0	0	1	0	0	0
SN097	2	7	3	125.5	18	1	1	0	1	1	1	0	1	1	0	1	1	1	1
SN098	2	6	3	117	15	1	1	0	1	1	1	0	1	0	0	1	1	1	0
SN099	2	7	3	121.5	20	1	1	0	1	1	1	0	0	0	1	0	1	1	0
SN100	1	6	3	119	22.5	1	1	0	1	1	1	0	1	0	1	0	1	0	0
SN101	1	6	3	118.5	18	1	1	0	1	1	1	1	1	0	0	1	1	1	1
SN102	2	6	3	116.5	18	1	1	0	1	0	1	0	1	0	1	1	1	0	0
SN103	2	7	3	125	23	1	0	0	1	0	1	0	1	0	1	1	1	1	0
SN104	1	6	3	118	22	1	1	0	1	0	1	1	1	1	0	1	1	0	1
SN105	2	8	3	129	26.5	1	1	1	1	1	1	1	1	1	1	1	1	1	1
SN106	1	9	3	141	34	1	1	1	1	1	1	1	1	0	1	1	1	1	1
SN107	1	8	3	130.5	28.5	1	1	0	1	1	1	0	1	0	0	0	1	1	1
SN108	2	9	3	138	42	1	1	0	1	0	1	1	1	1	0	1	1	1	0
SN109	2	8	3	132.5	26.5	1	1	0	1	1	1	0	1	1	0	1	1	0	0
SN110	2	7	3	124.5	19	1	1	0	1	1	1	1	1	0	0	1	1	0	1
SN111	2	6	3	116	15	1	1	0	1	1	1	1	1	0	0	1	1	0	0
SN112	2	7	3	123.5	21.5	1	1	0	1	1	1	0	1	0	1	1	1	0	1
SN113	1	9	3	147	42	1	1	0	1	1	1	0	0	0	1	1	1	1	0
SN114	1	7	3	123.5	30.5	1	1	0	1	1	1	1	1	0	1	1	1	1	0
SN115	2	9	3	132	31.5	1	1	0	1	1	1	0	1	0	0	1	1	0	0
SN116	1	9	3	131	30	1	1	1	1	1	1	1	1	1	1	1	1	0	1
SN117	1	9	3	134.5	31.5	1	1	0	1	1	1	1	0	0	0	1	1	0	0

SN118	1	7	3	125.5	25.5	1	1	0	1	0	1	1	1	0	0	1	0	1	0
SN119	2	9	3	130	29.5	1	1	0	1	1	1	1	1	0	0	1	1	1	0
SN120	2	6	3	116	22	1	1	0	1	1	1	1	1	0	0	1	1	1	0
SN121	1	9	3	145	44	1	1	0	1	1	0	0	1	0	1	0	1	1	0
SN122	2	6	3	119	18	1	1	0	1	1	1	0	1	0	0	0	1	0	0
SN123	2	8	3	122.5	21.5	1	1	0	1	1	1	1	0	0	1	1	0	1	1
SN124	1	6	3	121.5	29	1	1	0	1	1	1	0	1	0	1	1	0	0	0
SN125	2	7	3	121.5	23	1	1	0	1	0	0	1	1	0	0	1	1	0	1
SN126	2	7	3	121	24	1	1	0	1	0	1	0	1	0	0	1	1	1	0
SN127	2	6	3	117.5	22	1	1	1	1	0	1	1	0	1	1	1	1	1	1
SN128	2	7	3	127	33	1	0	0	0	0	0	0	0	0	0	0	0	0	0
SN129	2	6	3	122	28	1	1	0	0	0	1	0	0	0	0	1	1	1	1
SN130	1	8	3	133.5	27.5	1	1	0	0	0	1	1	1	0	0	1	0	1	0
SN131	2	6	3	120	25	1	1	0	1	1	1	1	1	0	1	1	1	0	1
SN132	1	6	3	115.5	19	1	1	0	1	1	0	0	0	0	0	1	1	1	0
SN133	2	8	3	133	22	1	0	0	0	0	0	0	0	0	0	0	0	0	0
SN134	1	7	3	130.5	33	1	0	0	1	0	1	0	1	0	0	0	1	0	0
SN135	2	9	3	127.5	24.5	1	1	0	0	0	0	0	1	0	0	1	1	0	0
SN136	1	9	3	135	23	1	1	0	0	1	1	0	0	0	0	1	1	1	1
SN137	1	9	3	130.5	27.5	1	1	0	0	0	0	0	0	0	0	0	0	0	0
SN138	2	6	3	120	28.5	1	1	1	0	0	0	0	1	1	0	0	1	1	0
SN139	1	6	3	121	26.5	1	1	0	0	0	0	0	1	0	0	1	0	0	0
SN140	1	9	3	141.5	41	1	1	0	0	1	1	1	0	0	0	0	0	0	0
SN141	2	8	3	130	28	1	1	1	1	1	1	1	1	1	1	1	1	1	0
SN142	2	6	3	127	19	1	1	0	0	0	0	0	1	0	0	0	0	0	0
SN143	1	7	3	129.5	27	1	1	0	1	0	0	1	0	0	0	0	1	0	0
SN144	1	6	3	119.5	21	1	1	0	0	1	1	0	1	0	0	1	1	0	0
SN145	1	8	3	126	25.5	0	1	0	0	0	1	0	0	0	1	1	0	1	0
SN146	1	7	3	123	29.5	1	1	0	1	1	1	1	1	0	0	1	1	1	1
SN147	2	7	3	131.5	24.5	0	1	0	0	0	1	0	0	0	0	1	0	1	0
SN148	1	7	3	125.5	24.5	1	1	0	1	1	1	1	1	0	0	1	1	0	1
SN149	2	7	3	119	23	1	1	0	1	1	1	1	0	0	0	1	0	0	0
SN150	1	9	3	138	31.5	1	1	0	1	1	1	1	1	1	0	1	1	0	0
SN151	2	7	3	127.5	19.5	1	1	0	1	0	1	0	1	0	0	1	0	0	0
SN152	2	8	3	138	35.5	1	1	0	0	0	1	0	1	0	0	1	1	1	0
SN153	2	6	3	123	21.5	1	1	1	1	1	1	1	1	0	1	1	1	1	0
SN154	1	7	3	129	25	1	0	0	1	0	1	0	0	0	0	0	0	0	0
SN155	1	9	3	132	27.5	1	1	0	1	0	0	1	0	0	0	0	1	0	0
SN156	1	6	3	125.5	21.5	1	0	0	1	0	1	0	0	0	0	0	0	0	0
SN157	2	7	3	125.5	21	1	0	0	1	0	0	0	0	0	0	0	0	0	0
SN158	2	9	3	140.5	38	1	1	0	1	0	0	1	1	0	0	1	1	1	0
SN159	1	6	3	115	22	1	1	1	0	1	0	0	1	0	0	1	0	0	0
SN160	2	6	3	112	18	1	0	0	0	1	0	0	0	0	0	0	0	1	0
SN161	2	6	3	119	18.5	1	1	0	1	0	1	0	1	0	0	1	0	0	0
SN162	2	7	3	117.5	18	1	0	0	1	0	1	1	1	0	0	1	1	0	1
SN163	1	6	3	125.5	29.5	1	0	0	1	0	1	0	1	0	0	0	1	0	0
SN164	2	9	3	128	26.5	1	1	0	0	0	0	0	0	0	0	0	0	0	0
SN165	2	8	3	132.5	30	1	0	0	0	0	0	0	0	0	0	1	1	1	0
SN166	2	9	3	131.5	30	1	1	0	1	1	1	1	1	1	0	1	1	1	1
SN167	1	8	3	130.5	31.5	1	1	0	1	1	1	1	0	0	0	1	0	0	0
SN168	2	9	3	131.5	27.5	1	1	1	0	1	1	1	0	0	0	0	0	1	1
SN169	1	8	3	135	32.5	1	1	0	0	1	0	1	1	0	1	0	1	1	0
SN170	1	8	3	130	25.5	1	1	0	0	1	1	0	1	0	0	1	1	0	0
SN171	2	6	3	126	29.5	1	0	0	1	0	1	0	0	0	0	0	0	0	0
SN172	2	8	3	135.5	37	1	1	1	1	1	1	1	1	1	1	1	1	1	1
SN173	1	6	3	124.5	27.5	1	1	0	1	0	1	0	1	0	0	1	0	0	0
SN174	1	8	3	137.5	44	1	1	0	0	0	0	0	0	0	1	1	1	0	0
SN175	2	8	3	134	36	1	1	0	0	0	1	0	0	0	0	1	1	1	0
SN176	2	7	3	127	24.5	1	1	0	0	0	1	0	0	0	0	1	1	1	0
SN177	2	8	3	138.5	29	1	1	0	0	0	1	0	1	0	0	1	0	0	0
SN178	1	7	3	121	26.5	1	1	1	1	1	1	1	1	0	1	1	1	1	1
SN179	1	8	3	133.5	36	1	1	0	1	0	0	1	0	0	0	0	1	0	0
SN180	2	6	3	116	18	1	0	0	1	0	0	0	0	0	0	0	1	0	0
SN181	1	9	3	141	40.5	1	1	0	1	1	1	1	1	1	1	1	1	1	0
SN182	2	9	3	139.5	40.5	1	1	1	0	1	1	1	0	1	1	1	1	1	1
SN183	2	9	3	135	29	1	0	0	1	0	1	0	1	0	0	0	1	0	0
SN184	1	7	3	121	25.5	1	1	0	1	1	1	1	0	0	0	1	0	0	0
XZ001	2	7	4	125	26	1	1	0	1	1	1	1	1	0	1	0	1	1	0
XZ002	2	8	4	129.5	26.5	1	0	0	1	0	1	0	1	0	0	0	1	0	0

XZ003	2	6	4	123.5	25.5	1	1	0	1	0	0	1	0	0	0	0	1	0	0
XZ004	1	8	4	126.5	24	1	1	0	1	1	1	1	1	0	0	0	1	0	0
XZ005	1	8	4	133.5	20.5	1	1	0	1	1	1	1	0	0	0	1	0	0	1
XZ006	2	8	4	132	26.5	1	1	0	1	1	1	1	0	0	0	0	0	0	1
XZ007	2	8	4	125	26.5	0	1	0	1	1	1	1	0	0	0	1	1	1	0
XZ008	1	6	4	122.5	29	1	1	0	0	1	1	1	1	0	0	0	1	1	1
XZ009	1	6	4	122	22.5	1	1	1	1	1	1	0	1	0	0	0	1	0	1
XZ010	2	9	4	144	28	1	1	0	0	0	0	0	1	0	0	1	1	1	0
XZ011	1	9	4	142	28	1	1	1	0	0	1	1	1	0	0	1	1	1	0
XZ012	2	7	4	126.5	24	1	1	0	0	0	1	1	1	0	0	1	1	1	1
XZ013	1	8	4	136	28.5	1	1	0	0	1	0	1	1	1	1	0	0	0	0
XZ014	2	8	4	135.5	33.5	1	1	1	0	1	0	1	1	0	0	1	1	1	1
XZ015	1	8	4	135	29.5	1	1	1	0	1	1	1	0	0	0	1	1	1	0
XZ016	1	9	4	139	28	1	1	1	1	0	1	1	1	1	0	1	1	0	1
XZ017	2	7	4	128.5	31	1	1	1	0	0	0	1	1	0	0	1	1	1	1
XZ018	1	7	4	133	26	1	1	0	0	1	1	1	1	1	0	1	1	1	0
XZ019	1	8	4	133	31	1	1	1	1	1	1	0	0	1	0	1	1	0	1
XZ020	1	7	4	127.5	25	1	1	1	1	1	1	1	1	0	0	0	1	0	1
XZ021	2	8	4	136	26.5	1	1	0	0	1	0	1	1	0	0	1	1	1	0
XZ022	2	7	4	129.5	25	1	1	1	0	1	0	0	1	0	0	1	0	0	0
XZ023	1	7	4	133	26	0	1	0	0	1	0	1	0	0	0	1	1	1	0
XZ024	1	6	4	127.5	29	1	1	0	1	1	1	0	0	0	0	1	1	1	1
XZ025	2	6	4	127	26	0	1	0	1	1	1	0	1	0	1	1	1	1	0
XZ026	1	9	4	136	36	1	1	0	0	1	1	1	1	0	0	1	1	1	0
XZ027	1	7	4	129.5	29	0	1	0	0	0	1	0	1	0	0	1	1	1	1
XZ028	2	8	4	133	30	1	1	0	1	0	0	1	1	0	0	1	0	1	0
XZ029	2	7	4	129.5	25.5	0	0	0	0	1	1	0	0	0	0	1	0	1	0
XZ030	1	6	4	126.5	26.5	0	1	1	1	0	0	0	0	0	0	1	1	1	0
XZ031	1	9	4	139	35	0	1	0	0	0	1	0	0	0	0	1	0	1	0
XZ032	2	9	4	144	30	1	1	1	0	1	1	1	0	0	0	0	0	1	1
XZ033	2	6	4	120	19	1	0	0	0	0	0	0	0	0	0	1	1	1	0
XZ034	2	9	4	146.5	35	1	1	0	0	0	1	1	0	0	0	1	1	1	1
XZ035	1	8	4	136.5	34.3	1	1	0	0	0	1	0	1	0	0	1	1	0	0
XZ036	1	6	4	124.5	23.5	1	1	0	0	0	1	0	0	0	0	1	1	1	1
XZ037	2	8	4	136	27.5	1	1	0	0	0	1	0	0	1	1	1	1	1	1
XZ038	2	9	4	144.5	32.5	1	1	0	1	0	1	1	0	1	0	1	1	1	0
XZ039	2	9	4	140.5	29.5	1	1	0	0	1	1	0	1	0	0	1	1	0	1
XZ040	1	7	4	137.5	34	1	1	0	0	1	0	0	1	1	0	0	1	0	1
XZ041	2	9	4	145.5	28.5	1	1	1	1	0	0	1	1	0	0	0	1	1	1
XZ042	2	9	4	149	34.5	1	1	0	0	0	0	1	1	1	0	1	1	1	0
XZ043	2	9	4	152.5	34.5	1	1	1	0	0	1	0	1	0	0	1	1	0	1
XZ044	2	9	4	137.5	26.5	1	1	0	0	1	1	1	1	0	1	1	1	1	1
XZ045	1	7	4	131.5	27.5	1	1	0	0	1	0	1	1	0	0	1	1	1	1
XZ046	2	8	4	126.5	24.5	1	1	0	0	1	1	1	1	0	1	1	1	1	1
XZ047	2	6	4	121	21.5	1	1	0	0	0	0	0	1	0	0	1	1	0	0
XZ048	1	9	4	139	39.5	1	1	1	1	1	1	0	1	1	1	1	1	1	1
XZ049	1	8	4	129	29	1	1	1	1	1	1	1	1	0	1	0	1	1	0
XZ050	1	6	4	117	20	1	0	0	1	1	0	0	0	0	0	0	0	0	0
XZ051	1	7	4	121	25.5	1	1	0	1	1	1	0	1	0	1	1	1	1	1
XZ052	2	8	4	127	26	1	1	0	1	0	1	0	1	0	0	1	0	0	0
XZ053	2	9	4	130	25	1	0	0	0	0	0	0	0	0	0	0	0	0	0
XZ054	1	6	4	121.5	19	1	0	0	0	0	0	0	1	0	0	0	1	0	1
XZ055	1	7	4	123	28	1	1	0	0	0	0	0	1	0	0	1	1	1	0
XZ056	2	7	4	128.5	23.5	1	1	0	0	0	1	1	1	0	0	0	1	0	1
XZ057	2	7	4	125.5	29	1	1	0	1	0	1	0	1	0	1	0	1	0	0
XZ058	1	6	4	112.5	20	1	1	0	0	0	1	0	1	0	0	1	0	0	0
XZ059	2	8	4	127.5	30.5	1	1	0	1	0	1	1	1	1	1	1	1	1	1
XZ060	2	7	4	128.5	24	1	1	0	1	0	0	1	1	0	0	1	0	1	0
XZ061	1	8	4	130	28	1	1	0	0	0	1	0	0	0	0	0	1	0	0
XZ062	2	6	4	123	29	1	1	0	0	0	0	0	0	0	0	1	0	0	0
XZ063	1	6	4	123.5	25.5	1	1	0	0	0	0	0	0	0	0	1	0	0	0
XZ064	1	6	4	122	20	0	0	0	0	0	0	0	0	0	0	0	0	0	0
XZ065	1	8	4	132	32	1	0	0	1	0	1	0	0	0	0	0	0	0	0
XZ066	2	6	4	123	19	1	1	0	1	0	0	1	0	0	0	0	1	0	0
XZ067	1	6	4	119.5	23	0	0	0	0	0	0	0	0	0	0	0	0	0	0
XZ068	2	7	4	127.5	29.5	1	0	0	1	0	1	0	0	0	0	0	1	0	0
XZ069	1	6	4	117	22	1	1	0	1	1	1	1	1	1	1	1	1	1	0
XZ070	2	7	4	129.5	37.5	1	1	0	0	0	1	0	1	0	0	1	1	0	0
XZ071	2	7	4	126.5	31.5	1	1	0	0	0	1	1	1	0	0	0	1	1	0

XZ072	1	7	4	125.5	23.5	1	1	0	1	1	1	1	0	0	0	1	1	1	0
XZ073	1	6	4	124	28	0	0	0	0	0	0	0	0	0	0	0	0	0	0
XZ074	1	7	4	120	24	1	1	0	1	0	1	1	1	1	0	1	1	1	0
XZ075	1	8	4	128	27.5	1	1	0	1	0	1	1	1	1	1	1	0	1	1
XZ076	1	8	4	144	29.5	1	1	1	1	1	1	1	1	0	1	1	1	1	1
XZ077	2	7	4	124.5	22	1	1	1	1	1	1	1	1	1	0	1	1	0	0
XZ078	1	6	4	117.5	24	1	1	0	0	1	0	1	1	1	0	0	0	0	0
XZ079	1	7	4	124.5	27	0	1	0	0	0	0	0	0	0	0	0	0	0	0
XZ080	2	9	4	142.5	36.5	1	1	0	0	0	1	0	1	0	0	1	1	1	0
XZ081	2	6	4	114	18	1	0	0	1	1	0	0	0	0	0	1	0	0	0
XZ082	1	7	4	124.5	24	0	0	0	0	0	0	0	0	0	0	0	0	0	0
XZ083	2	6	4	122	24	1	1	0	1	0	1	1	1	1	1	1	1	1	1
XZ084	2	8	4	123.5	25	1	1	0	0	0	1	0	1	0	0	0	1	0	0
XZ085	1	9	4	136	36	1	1	0	0	1	1	1	1	0	0	1	1	1	0
XZ086	2	8	4	126	27.5	1	1	0	0	0	1	0	0	0	0	0	1	0	0
XZ087	2	6	4	110	17.5	0	0	0	0	0	0	0	0	0	0	0	0	0	0
XZ088	2	8	4	121	23.5	1	1	1	1	1	1	1	1	1	0	1	1	1	1
XZ089	1	7	4	128.5	27.5	1	1	0	0	0	0	0	1	0	0	1	1	1	0
XZ090	2	6	4	119	18	0	1	0	0	0	0	0	0	0	0	0	0	0	0
XZ091	2	7	4	123.5	26.5	1	1	1	1	1	1	1	1	0	0	1	1	1	0
XZ092	2	6	4	111	16	0	0	0	0	0	0	0	0	0	0	0	0	0	0
XZ093	2	7	4	126.5	28	1	1	0	0	0	0	0	1	1	0	0	1	0	1
XZ094	2	7	4	126.5	20.5	1	1	0	0	0	1	0	1	0	0	1	0	0	0
XZ095	1	7	4	130.5	26.5	0	0	0	0	0	0	0	0	0	0	0	0	0	0
XZ096	1	7	4	126	24	1	1	0	0	1	0	0	0	0	0	1	0	1	1
XZ097	1	8	4	128.5	24	1	0	0	0	0	0	0	0	0	0	0	0	0	0
XZ098	1	8	4	129.5	28	1	1	0	0	0	1	0	1	0	0	1	1	0	1
XZ099	1	7	4	131.5	28	1	1	0	0	1	0	1	1	0	0	1	1	1	1
XZ100	1	8	4	136.5	34	1	1	0	0	0	0	0	0	0	0	0	0	0	0
XZ101	2	9	4	138.5	36.5	1	1	1	1	1	1	1	1	1	1	1	1	1	1
XZ102	2	9	4	141.5	34.5	0	1	0	0	0	1	0	1	0	0	1	1	1	1
XZ103	2	7	4	123.5	23	1	0	0	1	1	0	0	0	0	0	0	0	0	0
XZ104	2	9	4	135.5	35	1	1	1	1	1	1	1	1	0	1	1	0	1	1
XZ105	1	9	4	146	32	1	1	0	0	0	0	0	0	0	0	1	1	0	0
XZ106	1	8	4	134	33.5	1	1	1	0	1	1	0	1	1	0	1	1	1	0
XZ107	1	6	4	119.5	20	1	1	0	1	0	0	1	0	0	0	1	1	0	0
XZ108	2	9	4	137	38	1	1	0	0	0	0	0	0	0	0	0	0	0	0
XZ109	2	9	4	128.5	29.5	1	1	1	1	1	1	1	1	1	1	1	1	1	0
XZ110	1	6	4	118.5	21	0	0	0	0	0	0	0	0	0	0	0	0	0	0
XZ111	2	8	4	134.5	27	1	1	0	1	1	1	1	1	0	1	1	1	1	0
XZ112	2	7	4	125.5	23	1	1	0	1	0	0	1	0	0	0	0	1	0	0
XZ113	1	8	4	129	28.5	1	0	0	1	1	0	0	0	0	0	0	0	0	0
XZ114	1	7	4	127.5	28	1	1	0	0	1	0	0	0	0	0	1	1	0	0
XZ115	1	9	4	131	29.5	1	1	1	1	1	1	1	1	0	1	1	1	1	0
XZ116	2	8	4	127.5	25.5	1	0	0	0	0	0	0	0	0	0	0	0	0	0
XZ117	1	7	4	128.5	30	0	0	0	0	1	1	0	0	0	0	1	0	1	0
XZ118	2	7	4	125.5	25.5	1	1	0	0	1	0	1	1	1	0	0	1	1	0
XZ119	2	7	4	126	29	1	1	0	0	1	1	0	1	0	0	1	1	0	0
XZ120	2	8	4	139	31	1	1	0	0	1	1	1	0	0	0	0	0	0	0
XZ121	2	7	4	127.5	29	0	0	0	0	0	0	0	0	0	0	0	0	0	0
XZ122	2	6	4	120	22	0	1	0	0	0	0	0	0	0	0	0	0	0	0
XZ123	2	6	4	121	21	1	1	0	0	1	1	1	0	0	0	1	0	1	0
XZ124	2	7	4	125.5	23.5	1	1	0	0	1	1	1	0	0	0	0	0	0	0
XZ125	1	8	4	137	37	1	1	0	0	0	1	0	0	0	0	1	0	0	0
XZ126	2	9	4	136	34	1	1	0	1	0	0	1	0	0	0	1	0	1	1
XZ127	2	7	4	128.5	22.5	1	1	0	0	1	1	0	1	0	0	1	1	0	1
XZ128	1	8	4	128.5	29	1	1	0	0	1	0	0	1	0	0	1	1	1	0
XZ129	1	6	4	116	19	1	1	0	1	1	1	0	0	0	1	1	1	1	1
XZ130	2	6	4	122	21	1	1	0	0	0	1	0	0	0	0	1	1	1	0
XZ131	1	6	4	123.5	30.5	1	0	0	0	0	0	0	0	0	0	0	0	0	0
XZ132	1	8	4	136	44	1	0	0	1	0	0	0	0	0	0	0	0	0	0
XZ133	1	7	4	126.5	23.5	0	1	0	0	0	0	0	0	0	0	0	0	0	0
XZ134	1	6	4	122.5	30	1	1	0	0	0	0	0	0	0	0	0	0	0	0
XZ135	1	9	4	139	32	1	1	0	1	1	0	0	1	0	0	1	1	0	0
XZ136	1	6	4	121.5	24.5	1	1	1	0	0	0	1	0	1	1	1	1	1	0
XZ137	1	9	4	139.5	39.5	1	1	0	0	0	0	0	0	0	0	0	0	0	0
XZ138	2	8	4	135.5	27.5	1	0	0	1	1	0	0	0	0	0	0	0	0	0
XZ139	2	8	4	135.5	32.5	1	1	0	0	0	1	0	1	0	0	1	0	0	0
XZ140	1	7	4	127.5	26	1	1	0	1	0	0	1	1	0	0	1	1	0	0

XZ141	2	6	4	122	23.5	1	1	0	1	1	1	1	1	0	1	1	1	1	1
XZ142	1	9	4	137	30	1	1	0	0	1	0	0	0	0	0	1	0	1	1
XZ143	1	9	4	151	45	1	1	0	1	1	1	1	1	1	1	1	1	1	0
XZ144	2	7	4	125.5	23	1	1	0	0	1	1	1	1	0	1	1	0	0	0
XZ145	1	7	4	127.5	30.5	1	1	0	1	1	0	0	0	0	0	1	1	1	0
XZ146	1	9	4	130	24	1	1	0	0	1	1	0	1	0	0	1	1	0	0
XZ147	2	9	4	136	28.5	1	1	0	0	0	0	0	0	0	0	0	0	0	0
XZ148	1	9	4	136	32	1	1	0	1	1	0	1	1	1	1	0	0	1	0
XZ149	2	6	4	126	23	1	1	0	0	1	1	1	1	0	1	1	0	0	0
XZ150	1	6	4	123.5	28.5	1	1	0	0	0	0	0	0	0	0	0	0	0	0
XZ151	1	6	4	124.5	30	1	1	0	0	1	0	0	1	0	0	1	1	1	0
XZ152	2	8	4	139	31	1	1	0	1	1	0	0	1	0	0	0	1	0	1
XZ153	1	9	4	147	41.5	1	1	1	1	1	1	1	1	1	1	1	1	1	0
XZ154	1	6	4	124.5	27	1	1	0	0	0	0	0	0	0	0	0	0	0	0
XZ155	1	6	4	120.5	23	0	0	0	0	0	0	0	0	0	0	0	0	0	0
XZ156	1	6	4	119.5	20.5	1	1	0	0	1	1	1	0	0	0	0	0	0	0
XZ157	2	9	4	136.5	34	1	1	1	1	1	1	0	1	1	0	1	1	1	0
XZ158	1	7	4	115	22.5	1	1	0	0	1	1	0	1	0	0	1	0	0	1
XZ159	2	7	4	129.5	24.5	1	1	0	1	1	1	1	1	1	1	1	1	1	0
XZ160	1	7	4	125.5	24	1	1	0	0	1	1	0	0	0	0	0	1	0	0
XZ161	1	6	4	117	22	1	1	0	1	0	1	1	1	1	1	1	1	1	0
XZ162	1	9	4	144	36	1	1	1	1	1	1	1	1	1	1	1	1	1	0
XZ163	1	8	4	139.5	36	1	1	0	1	0	1	0	1	0	0	1	0	0	0
XZ164	1	6	4	119.5	22.5	1	1	1	0	1	1	1	0	1	1	1	1	1	0
XZ165	1	7	4	127.5	27	1	1	0	1	0	1	0	0	0	0	1	1	1	0
XZ166	1	7	4	125.5	22	0	1	0	1	0	0	1	0	0	0	0	1	1	1
XZ167	2	6	4	118	27	1	0	0	0	0	1	0	1	1	1	1	0	0	0
XZ168	2	8	4	126.5	27.5	1	1	0	1	1	1	1	0	1	0	1	1	0	1
XZ169	2	6	4	124	22.2	1	1	0	0	0	0	0	0	0	0	0	0	0	0
XZ170	2	8	4	140	34	1	1	1	1	1	1	1	1	0	1	1	1	1	1
XZ171	1	9	4	148	48.5	1	1	1	1	1	1	1	1	1	1	1	1	1	1
XZ172	2	8	4	133.5	25.5	1	1	0	1	0	1	1	1	1	0	1	0	0	0
XZ173	2	9	4	138.5	31	1	0	0	1	1	0	0	0	0	1	1	1	1	1
XZ174	2	7	4	125.5	28.5	1	1	0	0	0	0	0	0	0	0	1	0	0	0
XZ175	2	6	4	119	19.5	1	1	0	1	0	0	1	1	0	0	1	1	0	0
XZ176	1	6	4	120	19.5	1	1	0	0	0	0	0	1	0	0	1	1	0	0
XZ177	1	9	4	142	35	1	1	1	1	1	1	1	1	1	1	1	1	1	1
XZ178	2	9	4	134	25	1	1	1	1	1	0	1	1	1	0	1	1	1	0
XZ179	2	6	4	120	23	1	1	0	1	0	0	1	0	0	0	1	0	1	1
XZ180	1	8	4	136	31	1	1	0	0	1	1	1	0	0	0	1	0	1	0
XZ181	1	7	4	124.5	26.5	1	1	0	1	0	0	1	0	0	0	1	1	0	0
XZ182	2	6	4	112	19	0	1	0	0	1	0	0	0	1	0	0	0	0	0
XZ183	2	7	4	127	22.5	1	1	0	1	0	0	1	1	0	0	1	0	1	0
XZ184	2	8	4	137	29	1	1	0	0	0	1	0	1	0	0	1	0	0	0
XZ185	1	6	4	122.5	25.5	1	1	0	0	1	1	1	0	0	0	1	0	1	0
XZ186	1	6	4	116.5	21.5	1	1	0	0	1	0	0	0	0	0	0	0	0	0
XZ187	1	6	4	120	23	1	1	0	0	0	1	0	0	0	0	1	1	0	0
