# Supplementary figures and images for: Assessment of Validity of Children's Movement Skill Quotient (CMSQ) Based on the Physical Education Classroom Environment
Source: Biomed Res Int. 2020 Oct 16;2020:8938763. doi: 10.1155/2020/8938763 (PMC7586154; doi:10.1155/2020/8938763)

Photos of the CMSQ test video

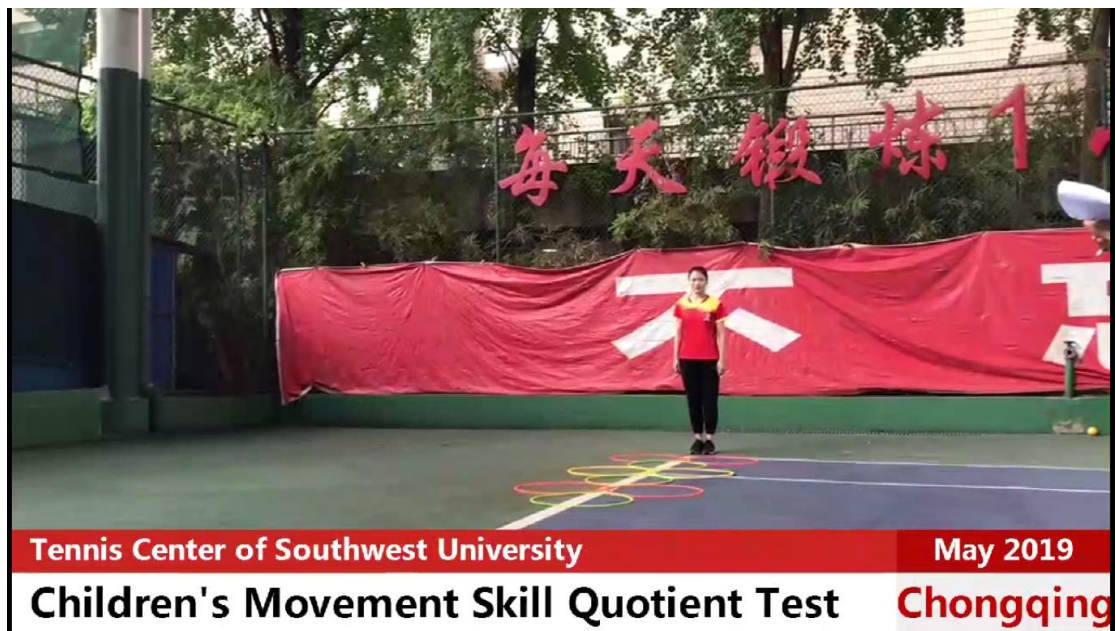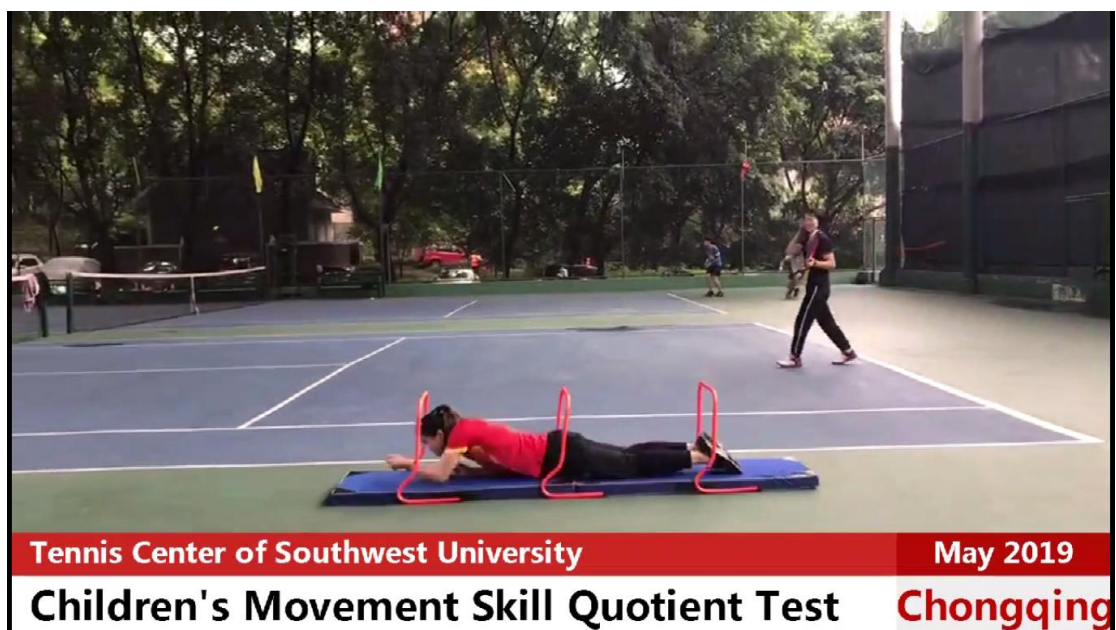

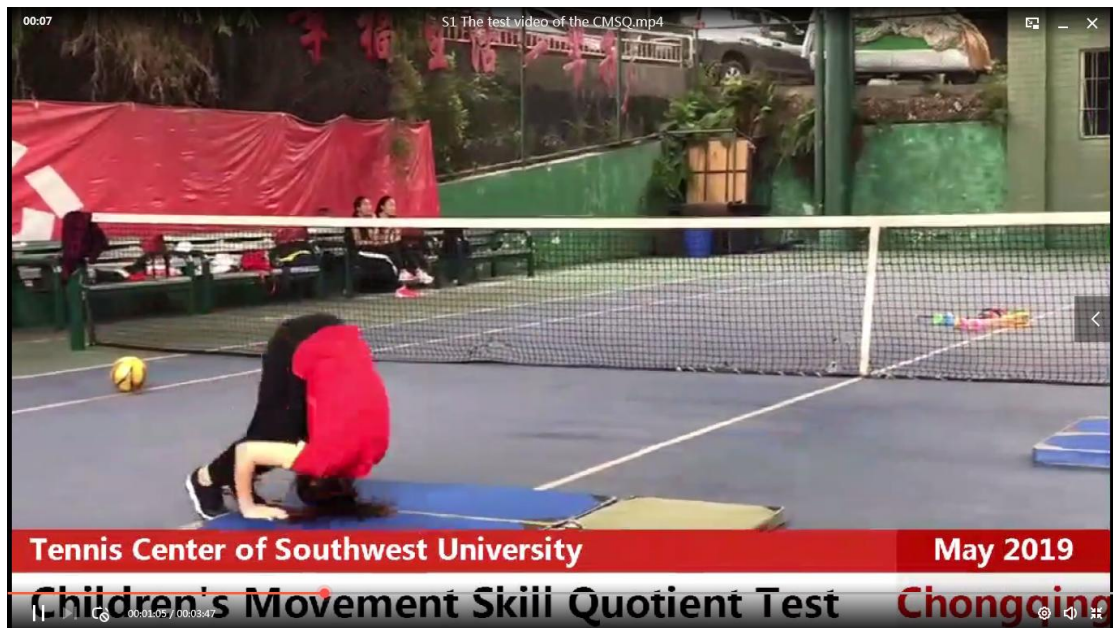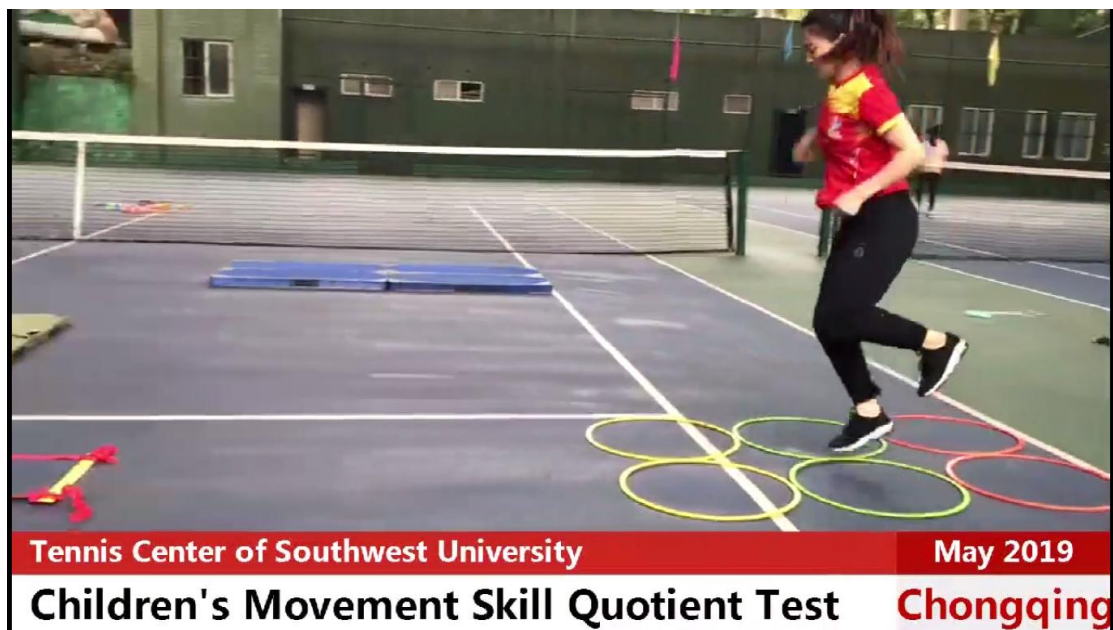

Supplement: Supplementary Materials — Table S1: the data of CMSQ (product). Video S1: the test video of the CMSQ. Video S2: rater assessment video of the CMSQ. [file 8938763.f1.zip › S2_Test video.pdf]
